# Exploring coral speciation: Multiple sympatric *Stylophora pistillata* taxa along a divergence continuum on the Great Barrier Reef

**DOI:** 10.1111/eva.13644

**Published:** 2024-01-26

**Authors:** Zoe Meziere, Iva Popovic, Katharine Prata, Isobel Ryan, John Pandolfi, Cynthia Riginos

**Affiliations:** ^1^ School of the Environment The University of Queensland St. Lucia Queensland Australia

**Keywords:** conservation, corals, demographic history, gene flow, population genomics, speciation continuum

## Abstract

Understanding how biodiversity originates and is maintained are fundamental challenge in evolutionary biology. Speciation is a continuous process and progression along this continuum depends on the interplay between evolutionary forces driving divergence and forces promoting genetic homogenisation. Coral reefs are broadly connected yet highly heterogeneous ecosystems, and divergence with gene flow at small spatial scales might therefore be common. Genomic studies are increasingly revealing the existence of closely related and sympatric taxa within taxonomic coral species, but the extent to which these taxa might still be exchanging genes and sharing environmental niches is unclear. In this study, we sampled extensively across diverse habitats at multiple reefs of the Great Barrier Reef (GBR) and comprehensively examined genome‐wide diversity and divergence histories within and among taxa of the *Stylophora pistillata* species complex. *S. pistillata* is one of the most abundant and well‐studied coral species, yet we discovered five distinct taxa, with wide geographic ranges and extensive sympatry. Demographic modelling showed that speciation events have occurred with gene flow and that taxa are at different stages along a divergence continuum. We found significant correlations between genetic divergence and specific environmental variables, suggesting that niche partitioning may have played a role in speciation and that *S. pistillata* taxa might be differentially adapted to different environments. Conservation actions rely on estimates of species richness, population sizes and species ranges, which are biased if divergent taxa are lumped together. As coral reefs are rapidly degrading due to climate change, our study highlights the importance of recognising evolutionarily distinct and differentially adapted coral taxa to improve conservation and restoration efforts aiming at protecting coral genetic diversity.

## 1 INTRODUCTION

The process of speciation, by which one population becomes two reproductively isolated species, is dynamic and involves genetic, geographical and environmental factors (Coyne & Orr, 2004; Nosil et al., 2017). Progression along the speciation continuum depends on the interplay between forces driving genetic divergence, such as genetic drift and selection, and forces driving genetic homogenisation, such as gene flow and recombination (Gourbière & Mallet, 2010). Over time, intrinsic and extrinsic incompatibilities accumulate between diverging taxa, reducing effective gene exchange and may eventually lead to complete speciation (Gourbière & Mallet, 2010; Roux et al., 2016; Westram et al., 2022).

With genomic data, we have unprecedented resolution to understand speciation dynamics and rigorously test long‐standing theories in natural populations (Campbell et al., 2018). In particular, genomic studies are increasingly revealing that strict allopatry is not necessary for speciation and that genetic differences can accumulate despite gene flow among diverging taxa (Feder et al., 2012). This is especially true in the marine environment, where there are few barriers to gene flow between incipient species (Faria et al., 2021; Potkamp & Fransen, 2019). The divergence process is often complex, relating to historical patterns of isolation, secondary contact and range dynamics. Using modelling approaches, we can sometimes differentiate among competing scenarios, such as secondary contact or ancient migration, and estimate the extent of gene flow during speciation (Sousa & Hey, 2013). Recent speciation studies have suggested that gene flow during speciation is heterogeneous across the genome, where loci contributing to adaptation or reproductive isolation are expected to experience reduced introgression and be more strongly differentiated (Ravinet et al., 2017; Wolf & Ellegren, 2017). Investigating genomic variability in differentiation for populations connected by varying levels of gene flow is therefore important to improve understanding of genomic semi‐permeability during speciation (Feder et al., 2012).

When divergent taxa arise in sympatry, we expect divergent selection on resources or habitat use to contribute to the evolution of reproductive barriers (Bolnick & Fitzpatrick, 2007; Schluter, 2009). Specifically, ecological adaptation in heterogeneous environments can promote assortative mating and the accumulation of genetic differences between locally adapted taxa, which can eventually lead to speciation within dispersal ranges (Potkamp & Fransen, 2019). Because gene flow can be pervasive during the transition from populations to reproductively semi‐isolated species, defining species boundaries can be ambiguous (Roux et al., 2016). By combining spatial and genomic information, species can be identified as groups of individuals forming distinct genotypic clusters that overlap in space (Mallet, 1995).

Scleractinian corals, the foundation species of coral reef ecosystems, are attractive yet largely underexploited systems to study speciation and environmental adaptation. With few impermeable physical dispersal barriers and steep environmental gradients characteristic of coral reefs (Hopley et al., 2007), speciation with gene flow could be likely. Although corals are long appreciated for their high biodiversity, the extent of such diversity and the mechanisms that have created it remain unclear. In fact, genomic studies are increasingly discovering divergent coral taxa not recognised by traditional taxonomy, often occurring within per generation dispersal distance (e.g. Bongaerts et al., 2017, 2021; Cooke et al., 2020; Fifer et al., 2022; Prada & Hellberg, 2021; Prata et al., 2022; Rippe et al., 2021; Rose et al., 2018; Sturm et al., 2020; Underwood et al., 2020). In many instances, genetic discontinuities coincide not only with depth (e.g. Prada & Hellberg, 2021; Prata et al., 2022; Rippe et al., 2021; van Oppen et al., 2018) but also with habitat exposure (e.g. Warner et al., 2015). However, most studies lack the appropriate sampling design to investigate associations between genetic discontinuities and specific environmental variables. Nevertheless, these coral species complexes represent exemplars of the speciation continuum and constitute compelling study systems to investigate the mechanisms driving diversification in the marine environment.

Resolving species boundaries and the processes driving diversification have direct conservation implications (Bickford et al., 2007; Hey et al., 2003). In particular, the prevalence of multiple genetically distinct taxa within nominal species suggests that coral species richness is often underestimated and that our understanding of species' geographical or environmental ranges might be inaccurate. Specifically, lumping multiple distinct taxa under the same species name will inflate population size estimates that could be used to make inferences about extinction risks (Dietzel et al., 2021; Muir et al., 2022). This lack of knowledge also impedes conservation spatial planning aiming to prioritise the protection of areas with high species richness or harbouring endemic species (van Oppen & Aranda Lastra, 2022). Finally, coral taxa that are both closely related and sympatric are likely to be differentially adapted to their environment and might therefore not have the same level of resilience facing environmental stressors. For example, thermal tolerance has been shown to vary between cryptic *A. hyacinthus* species (Rose et al., 2021) and between *Pocillopora* sister species (Burgess et al., 2021), suggesting that heatwaves might affect these taxa differentially. Corals are extremely vulnerable to global warming and restoration actions such as assisted gene flow in the form of larval seeding or colony outplanting are being considered to introduce putative heat‐resistant alleles into populations lacking those genetic variants (Quigley et al., 2019; van Oppen et al., 2017). In order to target appropriate populations, reefs and habitats, these interventions therefore rely on understanding of coral species identity and their ecological niches.

In this study, we examined genetic diversity within *Stylophora pistillata* (Esper, 1792) across the Great Barrier Reef (GBR), Eastern Australia. We identified distinct taxa and investigated their geographic distributions, divergence histories and environmental niche partitioning. *S. pistillata* is a well‐studied and common coral species with a brooding reproductive strategy allowing rapid larval settlement within hours from release (Fadlallah, 1983). Long thought to be widely distributed across the Indo‐Pacific region, molecular studies based on few mitochondrial and nuclear markers have previously identified four distinct and deeply divergent clades across this broad range (Flot et al., 2011; Keshavmurthy et al., 2013; Klueter & Andreakis, 2013): one homogeneous clade in the eastern Indian Ocean and Pacific Ocean and three clades restricted to the western Indian Ocean and Red Sea, with no geographic differentiation observed within clade. Using a reduced representation sequencing approach, additional taxa within the Red Sea clade have been discovered, with at least eight genetic groups identified (Buitrago‐López et al., 2023). In light of these previous findings, we hypothesised that *S. pistillata* might also represent a species complex on the GBR. In this study, we extensively sampled diverse habitats across the GBR and used reduced representation genomic sequencing to (i) identify divergent taxa within *S. pistillata*, (ii) investigate the extent of gene flow during speciation and (iii) characterise taxon‐specific environmental niche preferences. These results improve our understanding of coral diversity and shed light on the role of gene flow and environmental heterogeneity in coral speciation. We emphasise the importance of such findings for the conservation of vulnerable coral taxa in rapidly declining ecosystems.

## 2 MATERIALS AND METHODS

### 2.1 Sample collection

**FIGURE 1:**
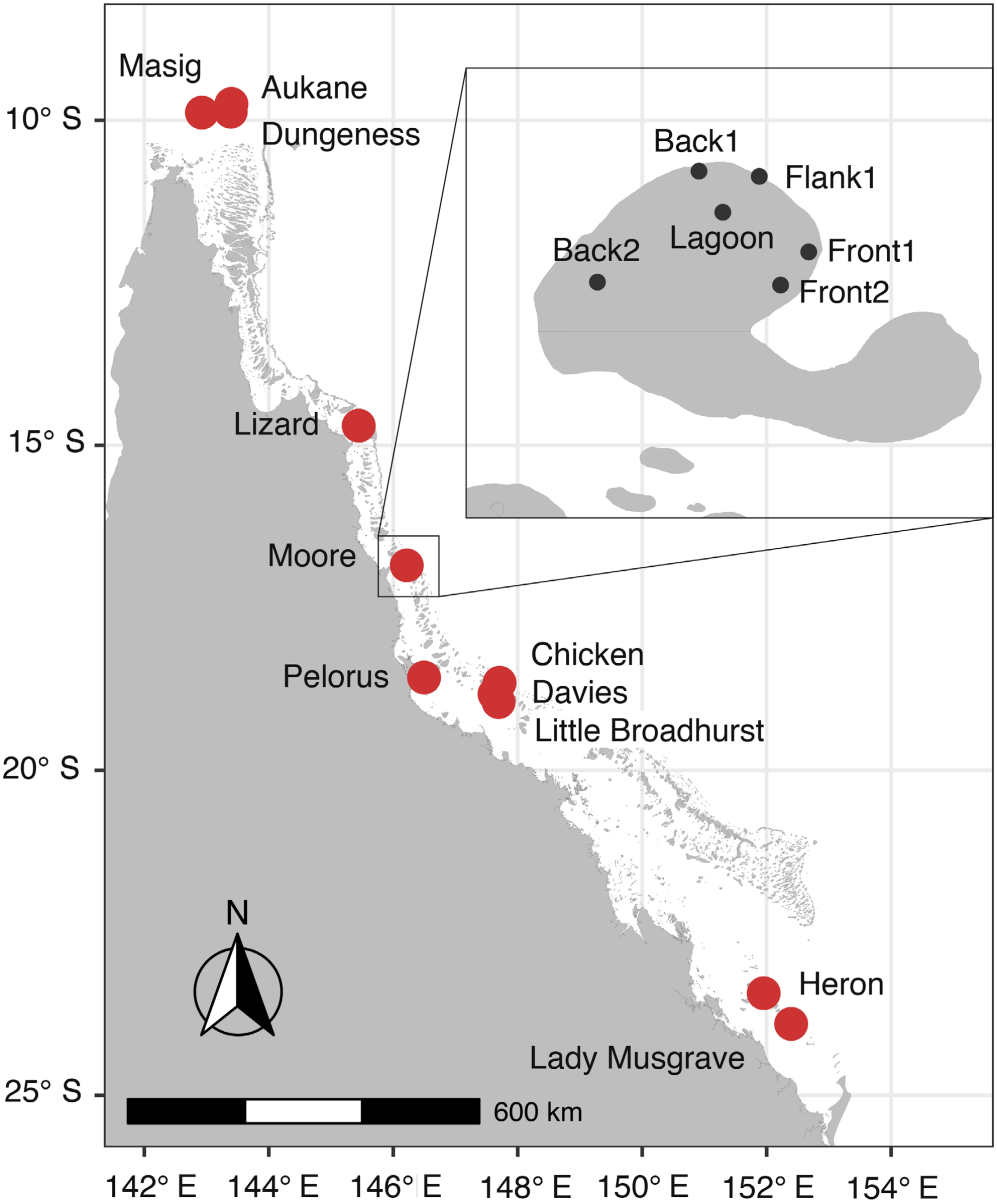
Map of the Great Barrier Reef showing the 11 reefs where adult *Stylophora pistillata* coral colonies were sampled. Inset shows the locations of the sampling sites at Moore Reef, although the two depths (5 and 15 m) at each site are not pictured. A similar sampling design was used at all reefs.

Adult colonies of *S. pistillata* were sampled using SCUBA (permit number G21/44774.1), from 11 reefs across the Great Barrier Reef (GBR), Eastern Australia (Figure 1, Table S1). At each reef, we sampled different habitats (back reef, front reef, lagoon and flank reef) and two depths were sampled at most sites (at approximately 5 and 15 m). This nested sampling design totals to 69 sites and we collected 434 coral colonies. Colonies were sampled using morphological criteria described in *Corals of the World* (Veron & Stafford‐Smith, 2000) to guide identification in the field. All colonies were photographed at the colony and corallite levels. Fragments, between 1 and 3 cm^2^, were preserved in 100% ethanol in the field and then kept at −20°C in the laboratory.

### 2.2 DNA extraction, library preparation and sequencing

Genomic DNA was extracted using the DNeasy QIAGEN kit according to the manufacturer's instructions. Agarose electrophoresis gels were run to visually assess the quantity and quality of the DNA extracted. Double‐digest restriction‐site‐associated DNA (ddRAD) libraries were prepared following the protocol described in Hereward et al., 2020. In brief, DNA plates of 96 individuals were normalised to 200 ng per sample and double digested with *MspI* and *PstI* enzymes before being ligated with a unique combination of forward and reverse barcodes for each sample. All 96 samples of each plate were then pooled in equimolar ratios, size selected to 300–400 bp on a Blue Pippin (Sage Science) and PCR amplified with added indices to differentiate libraries. Amplified libraries were then normalised and pooled together for sequencing. Sequencing was performed on two NovaSeq SP 300 lanes at the Australian Genome Research Facility (AGRF).

### 2.3 Processing and filtering of genomic data

Demultiplexing, filtering, clustering, alignment of reads and variant calling were performed in IPYRAD (Eaton & Overcast, 2020). We used standard parameters and mapped reads against the Great Barrier Reef *Stylophora pistillata* draft reference genome (GenBank genome assembly APGP_CSIRO_Spis_v1). Individuals with fewer than 1000 consensus reads (7 individuals) were removed for the final alignment. Then, further filtering of reads was performed in VCFTools (Danecek et al., 2011), following a two‐step process. First, we filtered for bi‐allelic sites, a minimum allele count (MAC) of 3, a minimum read depth of 5, a maximum read depth of 100 and a maximum of 50% missing data per site. Following this, individuals with more than 70% missing data (24 individuals) were removed. In addition, we identified clonal pair using the detect_clones.py script from P. Bongaerts (https://github.com/pimbongaerts/radseq) and kept one individual per pair, choosing the individual with the least amount of missing data. This dataset was then further filtered to allow only 5% missing data per site and a minimum allele frequency (MAF) of 0.01. Finally, SNPs in physical linkage were removed using PLINK (Purcell et al., 2007; http://pngu.mgh.harvard.edu/purcell/plink/) with a sliding window of 50 SNPs, 5 SNPs to shift window and a variance inflation factor (VIF) threshold of 2.

### 2.4 Identification of distinct taxa

To identify genetically distinct groups of individuals, we first performed a principal components analyses (PCA) (Pearson, 1901) with the glPca() function from the ‘adegenet’ package (Zheng et al., 2012) in R version 4.3.0 (R Core Team, 2023) using the first 10 PC axes. We then performed K‐means clustering on the PC axes loadings using the ‘stats’ R package (R Core Team, 2023) to confirm our visual grouping of individuals. To estimate individuals' ancestry proportions, we used ADMIXTURE (Alexander et al., 2009), with *K* ranging from 2 to 10 genetic groups, and we calculated cross‐validation errors and likelihoods for each *K*. To resolve geographic population structure from species‐level divergence, we also performed PCA and ADMIXTURE separately for each geographic region and for each identified genetic group. These analyses demonstrated the presence of five distinct genetic groups, referred thereafter as taxa.

### 2.5 Phylogenetic relationships

To examine taxa relationships, we generated species trees for each SNP using SNAPP in BEAST2 (Bryant et al., 2012) based on 10 individuals from each identified taxon. These 10 individuals were chosen randomly but were representative of the geographical range of each taxon. We used 250,000 MCMC generations, sampling every 100 steps. Tracer (Rambaut et al., 2018) was used to check the convergence of the chains and DensiTree (Bouckaert, 2010) to visualise the species trees.

To infer a phylogeny while allowing for the possibility of gene flow between taxa, we used TreeMix 1.13 (Pickrell & Pritchard, 2012) on the same subset of 10 individuals per taxon. This algorithm estimates population splits and tree topologies while modelling migration events between branches. We set a sliding window of 100 SNPs, used between 0 and 4 migration edges with 10 iterations for each and generated 100 bootstrap replicates.

### 2.6 Genetic divergence between taxa

Pairwise *F*_ST_ according to Weir and Cockerham (1984) was calculated using the pairwise.WCfst() function in the ‘adegenet’ R package, between populations from the same taxon and between populations from different taxa.

Genetic distances between all individuals sampled at the same reef were also calculated as the pairwise squared Euclidian distance using allelic data with the pairDist() function in the ‘adegenet’ R package. We used the frequency distribution of these genetic distances to infer the presence of sympatric genetic groups at each reef. If genetic groups co‐occur on the same reef, we expect to have two or more peaks in the distribution of genetic distances between all individual pairs, whereby individuals belonging to the same genetic group will form a peak at a low genetic distance and individuals belonging to the different genetic groups will form peaks at higher genetic distances (similar to the barcode gap, Hebert et al., 2003).

Because *F*_ST_ measures are affected by within‐populations levels of genetic variation, we also calculated the absolute sequence divergence (*D*_*XY*_) between taxa. We first used BCFtools mpileup (Danecek et al., 2021) to generate a VCF file containing variable and invariable sites. Variable sites were filtered using VCFTOOLS as described in Section 2.3 and we did not remove SNPs in physical linkage. We then used PIXY (Korunes & Samuk, 2021) to calculate *D*_*XY*_ in 1000 bp windows, which were averaged following the authors recommendations.

To investigate the relative proportions of shared and fixed SNPs between populations, we calculated *F*_ST_ at the locus level using the ‘pegas’ R package (Paradis, 2010). We focused on four allopatric population pairs showing different levels of genome‐wide divergence: Taxon2 TSMA–Taxon2 TSAU (global *F*_ST_ = 0.019), Taxon1 ONLI–Taxon2 TSMA (global *F*_ST_ = 0.19), Taxon4 CBHE–Taxon5 OCCH (global *F*_ST_ = 0.36) and Taxon1 OCCH–Taxon5 CBHE (global *F*_ST_ = 0.47). To investigate whether interspecific populations have fewer highly differentiated SNPs (SNP‐based *F*_ST_ > 0.5) in sympatry than in allopatry, we also investigated the *F*_ST_ distributions of two sympatric interspecific population pairs: Taxon4 CBHE–Taxon5 CBHE (global *F*_ST_ = 0.33) and Taxon1 CBHE–Taxon5 CBHE (global *F*_ST_ = 0.52).

### 2.7 Detection of hybridisation and demographic modelling of divergence histories

Three putative hybrid individuals were identified due to their intermediate position in the PCA space and their admixed ancestry in the ADMIXTURE analyses. We tested these individual as early‐generation hybrids using NewHybrids (Anderson, 2008) using Taxon1, Taxon2 and Taxon3 as possible parental species. The first 200 loci with highest *F*_ST_ values between the putative parental species were identified using the gl.nhybrids() function from the ‘dartR’ R package and NewHybrids was run on this dataset using 50,000 sweeps and 100,000 burn‐in and simulation of 6 genotype classes: parental1, parental2, F1, F2, backcross with parental1 and backcross with parental2.

To investigate divergence histories and obtain estimates of gene flow rates and divergence times among taxa, we used Diffusion Approximation of Demographic Inference (dadi) v.2.1.1 (Gutenkunst et al., 2009) in PYTHON v.3.6 using a workflow developed by *K*. Prata (https://github.com/kepra3/kp_dadi). We tested 10 population pairs, consisting of 6 sympatric interspecific population pairs and 4 allopatric intraspecific population pairs to test the strength of gene flow and estimate divergence times between and within taxa. We defined a population as all the individuals from a specific taxon from a specific reef, and groups of individuals were defined as sympatric if they co‐occurred on the same reef. For the interspecific population pairs, we modelled five scenarios: divergence in isolation with no gene flow (SI), divergence with symmetric homogeneous gene flow (IM), divergence with symmetric heterogeneous gene flow (IM2M), secondary contact with symmetric homogeneous gene flow (SC) and ancient symmetric homogeneous gene flow (AM). For all intraspecific population pairs, we tested three scenarios: SI, IM and IM2M. Models with homogeneous gene flow assume constant gene flow across all loci, while the model with homogeneous gene flow assumes that a proportion of loci experience reduced gene flow (Figure S7).

For each population pair, we created a folded joint allele frequency spectrum (JAFS) by projecting down each population to maximise the number of SNPs and masking singletons and doubletons due to their high‐sequencing error rates and filtering in previous steps. For each model, we searched the parameter space by performing three rounds of manual optimisation of three‐, two‐ and onefold permutations, with at least 30 runs per round. This allowed us to search the parameter space adequately by choosing starting parameters from their lower to upper bounds, and then searching around the optimised peaks. The best fit for each model was chosen based on the AIC and log‐likelihood. A total of 100 bootstraps were performed on the best‐fit parameters and likelihood ratio tests were undertaken to choose the best model for each dataset. We then used the Fisher Information Matrix to estimate parameter standard deviations.

Finally, we converted the parameter values obtained by dadi (*θ*, ν1_dadi_, ν2_dadi_, *M*_dadi_ and *T*_dadi_) into divergence times in years, effective population sizes in number of individuals and gene flow rates in number of migrants between populations per generation. First, we calculated the total sequence length:

$$L=\frac{{\mathrm{SNP}}_{\text{dadi}}\times \text{Number of}\;\mathrm{RAD}\;\text{tags}\times \text{lengh of}\;\mathrm{RAD}\;\text{tags}}{{\mathrm{SNP}}_{\text{original}}},$$

where SNP_dadi_ is the number of SNPs retained for the dadi analyses after projection and SNP_original_ is the number of SNPs originally detected.

We then calculated ancestral population sizes (*N*_ref_, in number of individuals), population sizes of Population1 and Population2 (ν1 and ν2, in number of individuals), divergence times (*T*, in years), migration rates (*m*, in fraction of migrant individuals every generation) and gene flow rates (*M*_21_ and *M*_12_, the number of migrants per generation from Population2 to Population1 and from Population1 to Population2, respectively), using the following conversions:

$$N_{\mathrm{ref}}=\frac{\theta }{4\times \mu \times L}$$

$$\nu 1=\nu 1_{\text{dadi}}\times N_{\mathrm{ref}}$$

$$\nu 2=\nu 2_{\text{dadi}}\times N_{\mathrm{ref}}$$

$$m=\frac{M_{\text{dadi}}}{2\times N_{\mathrm{ref}}}$$

$$T=T_{\text{dadi}}\times 2\times N_{\mathrm{ref}}\times T_{\mathrm{gen}}$$

$$M_{21}=m\times \nu 1$$

$$M_{12}=m\times \nu 2$$

We used a mutation rate of *μ* = 1.2 × 10^−8^ mutations per base per generation, which has been estimated for Acroporidae corals (Zhang et al., 2022) and a generation time *T*_gen_ = 3 years, as *Stylophora pistillata* colonies from the Red Sea have been described to be reproductive as young as 2 years old (Rinkevich & Loya, 1979). We acknowledge that these are approximated estimates.

### 2.8 Ecological niche differentiation between taxa

To determine the relative contribution of environmental variables to the partitioning of genetic diversity and to explore whether taxa occupy different environmental niches, we used a partial redundancy analysis (pRDA) (Legendre & Legendre, 2012) performed in the ‘vegan’ R package. For our response matrix consisting of individual allele frequencies, we removed missing sites (558 retained SNPs) and used Hellinger's transformation. For the explanatory matrix, we considered seven environmental variables: ‘Depth’, ‘Temperature mean’, ‘Temperature daily range’, ‘Current speed mean’, ‘Current speed median’, ‘Light intensity mean’ and ‘Water clarity mean’, obtained for every sampling site via eReefs models (http://ereefs.ais.gov.au/ereefs‐aims) (See Table S2 for more details on environmental data and acquisition). These environmental variables were selected because we predicted depth, temperature, exposure and light availability to explain species distributions, and they are not colinear (Pearson's correlation coefficient <0.70, Figure S9). All environmental variables were centred and standardised. To reduce spatial autocorrelation, we computed distance‐based Moran's eigenvector maps (dbMEM) in the ‘adespatial’ R package and used MEM1, MEM2 and MEM3 to condition the RDA. We used forward selection to simplify the model, and all variables except ‘Temperature daily range’ were retained. Significance was tested by permutation (100,000 steps) and results were visualised using the first two RDA axes. We used adjusted *R* ^2^ values to estimate the proportion of variance explained by each variable and their combinations (Peres‐Neto et al., 2006).

## 3 RESULTS

### 3.1 Processing and filtering of genomic data

After initial filtering of RAD loci, we retrieved 399,449 loci and 538,512 SNPs. Using a 99% genetic similarity threshold, 14 clonal pairs were identified and 1 individual per clonal pair was retained for all analyses. After stringent filtering and LD pruning of variants, our final dataset contained 5332 SNPs and 379 individuals.

### 3.2 Identification of distinct taxa

**FIGURE 2:**
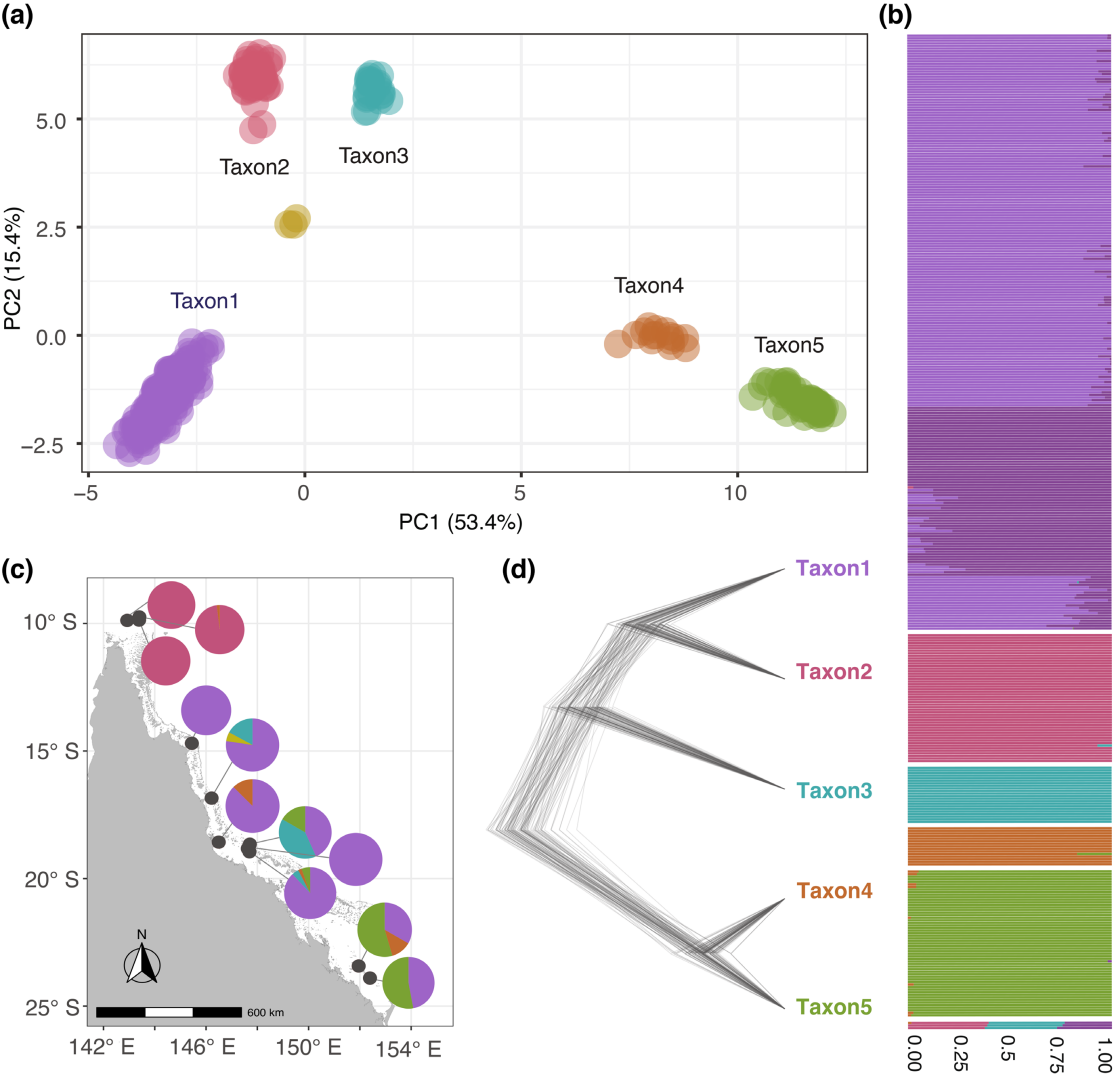
Population genomic structure and evolutionary relationships between taxa of the *Stylophora pistillata* species complex. (a) PC1 and PC2 from a principal component analysis showing five distinct taxa; (b) ancestry proportions from an ADMIXTURE analyses with *K* = 6 differentiating the five taxa. Each individual is represented as a horizontal line; (c) map showing the proportion of individuals belonging to each taxon at all sampling sites. Each colour represents a different taxon and pie charts are the proportions; and (d) SNAPP species tree supporting the five taxa and illustrating their phylogenetic relationships.

To visualise and understand the structuring of genetic variation among the 379 *S. pistillata* individuals, we first used PCA. This ordination indicated tight grouping of the individuals into five distinct groups, hereafter referred to as Taxon1, Taxon2, Taxon3, Taxon4 and Taxon5, which was confirmed by a K‐means algorithm searching for five groups in the data (Figure 2a). PC1 explained most of the variation (53.4%) and separated all five taxa. We examined the first 10 PC axes and found that the first 4 PC axes capture this structuring (Figure S2), which is consistent with theoretical work indicating that K‐1 PC axes capture the population structure of *K* effective populations (Patterson et al., 2006). We also noted an additional group of only three individuals, positioned among Taxon1, Taxon2 and Taxon3, and these individuals cluster together on subsequent PC axes.

To assign ancestry probabilities and detect potential admixture between groups, we ran ADMIXTURE with different values of *K*. At *K* = 2, Taxon4 and Taxon 5 separated from Taxon1, Taxon2 and Taxon3; at *K* = 3, Taxon1, Taxon2 and Taxon3 formed separate clusters; at *K* = 4, signals of population differentiation within Taxon1 were evident and Taxon2 and Taxon3 were re‐assigned the same ancestry proportions; and at *K* = 5, Taxon2 and Taxon3 formed separate clusters (Figure S1). Finally at *K* = 6, we found almost full assignment of each individual, despite population structure within Taxon1, consistent with the PCA results (Figure 2b, Figure S2). From the cross‐validation errors and the log‐likelihoods, it was not clear which *K* value was optimal (Figure S3). This can be explained by the very strong geographic population structure within Taxon1, which has the largest number of individuals and the largest geographic span, forcing the ADMIXTURE algorithm to show structuring within Taxon1 before other taxa despite the deeper divergences among taxa (Puechmaille, 2016). The three individuals forming an additional group on the PCA also showed a three‐way admixture pattern, contributed by Taxon1, Taxon2 and Taxon3, which is consistent with their position on the PCA. These individuals form a distinct group at *K* = 10 after intraspecific geographic population structure is detected within Taxon1 and within Taxon5, the most represented taxa in the full dataset (Figure S1). As explained above, the small number of individuals from this additional genetic group might be hindering their assignment as a distinct group by ADMIXTURE prior to the resolution of intraspecific structure in the analysis.

Analyses of taxon‐specific geographic distributions revealed that, apart from Taxon2 which is restricted to the Torres Strait, all the other taxa occur at multiple reefs. Specifically, Taxon1 is found in the South, Central and North GBR; Taxon3 is found in the Central and North GBR; Taxon4 is found in the South, Central GBR and in the Torres Strait; and Taxon5 is found in the South and Central GBR (Figure 2c). Additionally, taxa are sympatric at the reef and site level (Figure 2c).

Phylogenetic analyses recovered the five taxa and all SNAPP species trees had a consistent topology. Congruent with the position of the five clusters on the PCA, species trees revealed Taxon1 and Taxon2 to be sister taxa and to be more closely related to Taxon3 than to Taxon4 and Taxon5 which are also sister taxa (Figure 2d).

In addition, we performed PCA and ADMIXTURE analyses on each identified taxon separately and found clear geographic population structure within all of them (data not presented). This confirms that the results presented in Figure 2 reflect an interspecific level of divergence.

### 3.3 No evidence of recent hybridisation among taxa

With Taxon1 and Taxon2 or Taxon2 and Taxon3 set as possible parental species, NewHybrids analyses assigned the three putative hybrid individuals as one of the parental species. When Taxon1 and Taxon3 were set as the possible parental species, assignments were mixed, with one individual identified as having some probability of being a backcross (Figure S4). These individuals were dropped from further analyses. Consistent with negligible hybridisation, Treemix did not support improvements to the species tree with the addition of migration events, with an *f* value of 0.99 with zero migration edges (Figure S5). PCA and ADMIXTURE analyses are sensitive to unbalanced sample sizes (McVean, 2009; Puechmaille, 2016) and we hypothesise that these three individuals belong to a distinct taxon that we have not well sampled despite the assignment of mixed genetic ancestries. Due to the ambiguity in species assignment, we excluded these three individuals from downstream analyses.

### 3.4 Genetic divergence among taxa

To confirm the existence of multiple distinct sympatric genetic taxa at a given reef, we calculated the genetic distance between all pairs of individuals from the same reef and visualised them as frequency distributions. In all cases, we found at least two peaks, separated by significant gaps in frequency, representing the genetic distance between pairs of individuals belonging to the same taxon and pairs of individuals belonging to distinct taxa (Figure S6).

To investigate genetic differentiation between taxa, we estimated genome‐wide *F*_ST_ between all populations. *F*_ST_ between interspecific populations ranged from 0.19 (Taxon1 OCCH–Taxon2 TSAU) to 0.54 (Taxon1 CBHE–Taxon5 OCCH), which was significantly less than *F*_ST_ values between intraspecific populations, which ranged from 0.0099 (Taxon1 OCDA–Taxon1 OCLB) to 0.16 (Taxon5 OCCH–Taxon5 CBHE) (Figure 4a). These estimated *F*_ST_ values created a gradient in genetic differentiation from poorly differentiated populations to highly divergent populations. *D*_*XY*_ estimates between taxa ranged from 0.0074 (Taxon4–Taxon5) to 0.0099 (Taxon1–Taxon4) (Table 1), suggesting that *S. pistillata* taxa have on average less than 1% sequence divergence per base pair.

**TABLE 1:** Genetic divergence estimates (*D*_*XY*_, *F*_ST_) and results from the best fit demographic model (divergence with heterogeneous symmetric gene flow) performed in dadi on selected populations.

Level	Taxa pair	Population pair	*F*_ST_	*D*_*XY*_	Demographic modelling results									
					*N*_ref_	𝛎_1_	𝛎_2_	*m*	*m*_e_	*M*_21_	*M*_12_	*T*	*P*	*Q*
Intraspecific	Taxon2	T2TSAU–T2TSMA	0.019		4548	1027	6194	1.0e‐3	2.5e‐4	10.6	6.4	0.037	0.94	0.056
	Taxon3	T3OCCH–T3ONMO	0.081		43,561	81,787	128,577	1.5e‐5	1.4e‐6	1.3	2.0	0.45	0.94	0.060
	Taxon5	T5CBLM–T5CBHE	0.088		47,903	70,878	59,012	2.7e‐5	7.4e‐6	1.9	1.6	0.37	0.90	0.10
	Taxon1	T1CBHE–T1ONLI	0.15		27,679	31,020	127,982	1.0e‐5	5.2e‐6	0.32	1.35	0.38	0.88	0.12
Interspecific	Taxon1–Taxon2	T1ONLI–T2TSAU	0.19	0.0085	43,026	134,733	156,535	3.0e‐6	1.5e‐8	0.41	0.47	0.44	0.91	0.090
	Taxon4–Taxon5	T4CBHE–T5CBHE	0.33	0.0095	32,228	91,660	58,706	4.2e‐6	1.5e‐8	0.39	0.25	0.42	0.89	0.11
	Taxon1–Taxon3	T1ONMO–T3ONMO	0.33	0.0093	15,843	143,123	148,824	5.4e‐7	5.4e‐8	0.078	0.080	0.89	0.78	0.22
	Taxon1–Taxon3	T1OCCH–T3OCCH	0.34	0.0093	26,159	119,046	113,869	1.3e‐6	8.8e‐8	0.16	0.15	0.54	0.82	0.18
	Taxon1–Taxon5	T1CBLM–T5CBLM	0.52	0.0091	271,224	110,669	89,702	7.3e‐8	1.8e‐8	8.1e‐3	6.6e‐3	0.66	0.72	0.28
	Taxon1–Taxon5	T1CBHE–T5CBHE	0.52	0.0091	8282	100,038	93,345	2.4e‐7	6.0e‐8	0.024	0.022	0.92	0.72	0.28

*Note*: 𝛎_1_ and 𝛎_2_ are the estimated population sizes, in number of individuals; *m* is the estimated heterogeneous symmetric migration rate between populations every generation; *m*_e_ is the reduced migration rate between populations every generation; *M*_21_ and *M*_12_ are the estimated gene flow rates, in number of migrants from Population2 to Population1 and from Population1 to Population2, respectively, every generation; *T* is the estimated divergence time, in millions of years; *P* is the proportion of the genome evolving neutrally; and *Q* is the proportion of the genome experiencing reduced gene flow.

**FIGURE 3:**
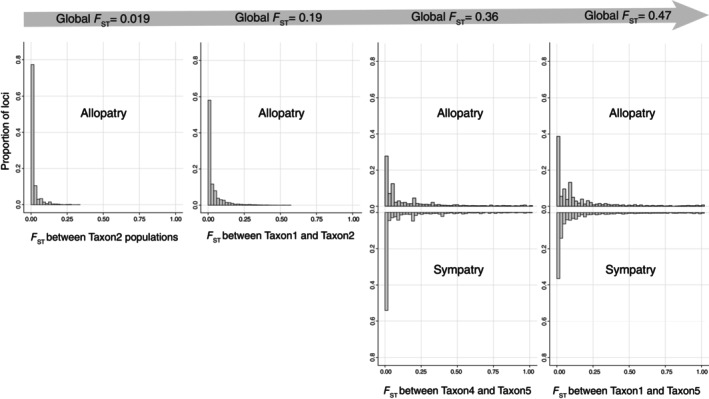
Distributions of locus‐level *F*_ST_ values in population pairs of *Stylophora pistillata* taxa on a spectrum of genetic divergence. For Taxon4–Taxon5 and Taxon1–Taxon5 comparisons, *F*_ST_ distributions between sympatric populations and between allopatric populations are presented. From left to right, the focal populations are as follows: Taxon2 TSMA–Taxon2 TSAU (allopatric; global *F*_ST_ = 0.019), Taxon1 ONLI–Taxon2 TSMA (allopatric; global *F*_ST_ = 0.19), Taxon4 CBHE–Taxon5 OCCH (allopatric; global *F*_ST_ = 0.36), Taxon4 CBHE–Taxon5 CBHE (sympatric; global *F*_ST_ = 0.33), Taxon1 OCCH–Taxon5 CBHE (allopatric; global *F*_ST_ = 0.47) and Taxon1 CBHE–Taxon5 CBHE (sympatric; global *F*_ST_ = 0.52).

In addition, we found SNP‐based *F*_ST_ distributions to vary depending on the global divergence level between populations (Figure 3). As global divergence increases between populations or taxa, we observed more highly differentiated loci (*F*_ST_ >0.5) and an increase in maximum *F*_ST_, from 0.3 within Taxon2 populations to 1 (fixation) between Taxon1 and Taxon5. Additionally, *F*_ST_ distributions were similar between allopatric and sympatric interspecific population pairs.

### 3.5 Demographic modelling of divergence histories

**FIGURE 4:**
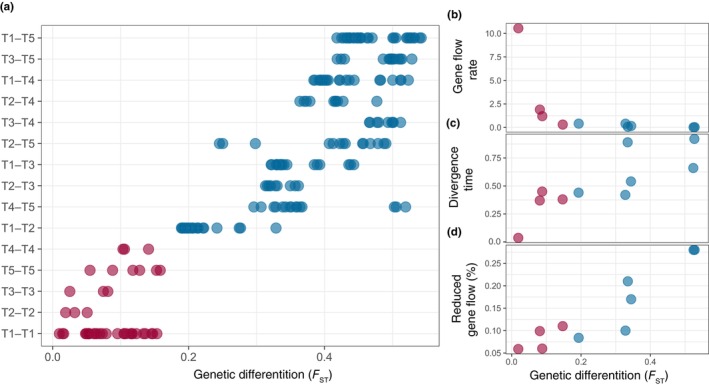
Genetic divergence of the *Stylophora pistillata* species complex. (a) Genetic differentiation between populations calculated as pairwise *F*_ST_, both within taxa (red dots) and between taxa (blue dots). (b) Gene flow rates (number of new migrant individuals in each generation), (c) divergence times (in million years) and (d) proportion of loci experiencing reduced gene flow between focal populations used for dadi demographic modelling, plotted against *F*_ST_.

To differentiate between speciation scenarios and to obtain estimates of gene flow rates and divergence times, we used demographic modelling in dadi. After examination of likelihoods, AIC and residuals of the modelled JAFS against the original data, we found that models with gene flow were a better fit than the SI model with no gene flow for all datasets (Table 1, Tables S3 and S4, Figure S8). For 6 of 10 population pairs, the IM2M model was favoured. For two population pairs (Taxon5 CBLM–Taxon5 CBHE and Taxon3 OCCH–Taxon3 ONMO), the IM and IM2M models had the best fit and had the same likelihood or an AIC difference smaller than 1. For the most divergent population pairs (Taxon1 CBLM–Taxon5 CBLM and Taxon1 CBHE–Taxon5 CBHE), we could not differentiate among all divergence models with gene flow (Table S4). When two or more models had similar fit to the data, the estimated parameters were consistent across models. We therefore present the estimated parameters for the IM2M models for all populations (Figure 4, Table 1). After converting parameter values from dadi, we found divergence times between taxa to vary between 0.42 (Taxon4 CBHE–Taxon5 CBHE) and 0.92 (Taxon1 CBHE–Taxon5 CBHE) million years and gene flow rates to vary between 0.006 (Taxon1 CBLM to Taxon5 CBLM) and 0.47 (Taxon1 ONLI to Taxon2 TSAU) migrant individuals per generation. In comparison, intraspecific population pairs had more recent divergence times between 0.037 (Taxon2 TSMA–Taxon2 TSAU) and 0.38 (Taxon1 CBHE–Taxon1 ONLI) million years and higher gene flow rates between 0.32 (Taxon1 ONLI to Taxon1 CBHE) and 10.6 (Taxon2 TSMA to Taxon2 TSAU) migrants per generation (Figure 4a,b, Table 1). Across all population pairs, the proportion of loci experiencing reduced gene flow varied from 6% (intraspecific, Taxon2 TSAU–Taxon2 TSMA) to 28% (interspecific, Taxon1 CBLM–Taxon5 CBLM and Taxon1 CBHE–Taxon5 CBHE). Overall, *F*_ST_ estimates were positively correlated with divergence times, negatively correlated with gene flow rates and positively correlated with the proportion of loci experiencing reduced gene flow (Figure 4d, Table 1).

### 3.6 Ecological differentiation between taxa

We performed a partial RDA to investigate how seven environmental variables correlate with genetic variation and whether genetically divergent taxa occupy different environmental niches. Forward selection on the explanatory matrix selected six environmental variables (Table S5) and collectively, these variables explained 12.04% of the variance in individual allele frequencies (adjusted *R* ^2^). The first three RDA axes were significant and RDA1 and RDA2 summarised 10.10% and 2.18%, respectively, of the variance (*F* = 42.09, *p* = 0.001; *F* = 9.05, *p* = 0.001; respectively).

**FIGURE 5:**
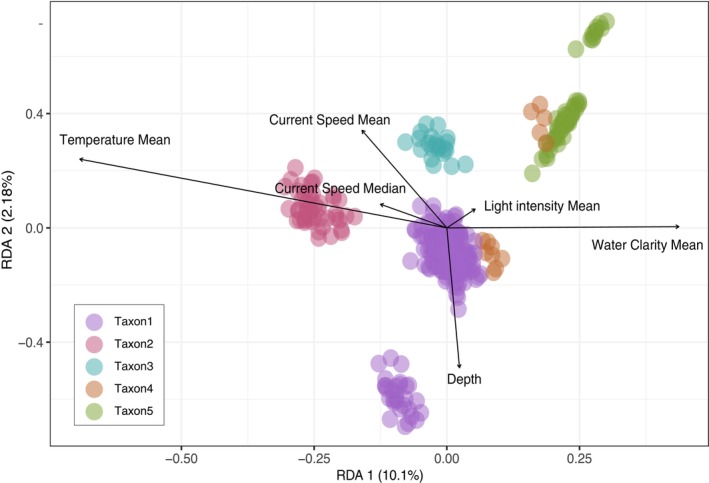
Ordination plot of partial redundancy analysis (pRDA) showing the relative contribution of environmental variables to the genetic variation among all *Stylophora pistillata* individuals, controlling for the effect of space (using dbMEM spatial vectors) as a function of environmental variables. Individuals are represented as circles coloured by taxon identity and environmental variables are represented as arrow vectors.

Taxon2 was segregated by the significant and positive effect of higher temperature mean, whereas Taxon3 was strongly associated with higher current speed. Some Taxon4 individuals seemed to be associated with higher water clarity. Taxon1 was associated with the depth gradient, which seemed to partition this taxon into two groups (Figure 5).

## 4 DISCUSSION

Using genome‐wide sequence data, we found that one of the most intensively studied corals, *Stylophora pistillata*, consists of five distinct taxa distributed across the Great Barrier Reef. With varying levels of genetic differentiation, these taxa appear to be at different stages along the speciation process and have likely speciated without hard geographic barriers to gene flow. We found that gene flow is heterogenous across genomes, and that differentiation among taxa is similar in sympatry and in allopatry, suggesting that reproductive barriers are firm at least for some parts of the genomes. We inferred Pleistocene divergence times and low levels of recent gene flow among taxa, also pointing to some level of reproductive isolation. Evidence of niche partitioning indicates that the environment helps maintain species boundaries and that environmentally associated selection might have played a role in *Stylophora* divergence history. These results add to the growing body of literature finding that coral diversity is vastly underestimated, which has direct implications for understanding species‐specific resilience to environmental changes.

### 4.1 Five taxa on a divergence continuum within ‘*Stylophora pistillata*’

Genomic data are increasingly revealing genetic discontinuities within corals species (e.g. Bongaerts et al., 2017; Cooke et al., 2020; Fifer et al., 2022; Matias et al., 2022; Prada & Hellberg, 2021; Prata et al., 2022; Rippe et al., 2021; Rose et al., 2018; Sturm et al., 2020; Underwood et al., 2020). Using reduced representation (RAD) genomic sequencing of 379 *S. pistillata* individuals from the Great Barrier Reef (GBR), we found five distinct taxa (Figure 2a,b). Taxon1, Taxon3, Taxon4 and Taxon5 have wide geographic distributions across the GBR and are sympatric at multiple reefs (Figure 2c). These results suggest that although taxa have the opportunity for interbreeding, exogenous and/or endogenous forces are maintaining their genetic distinctiveness. Following the genotypic cluster species definition, which emphasises genetic distinctiveness in sympatry (Mallet, 1995), these taxa should be considered different species. A distinct taxon, Taxon2, was exclusively found in the Torres Strait, where only one individual from another taxon (Taxon4) was sampled. The apparent allopatry between Taxon2 and other taxa makes it impossible to evaluate reproductive isolation. It is unlikely that Taxon2 occurs on the GBR, as we sampled extensively across diverse habitats and further genetic work, especially in Papua New Guinea and the Coral Triangle, would likely extend its known range.

Speciation is a continuous process, and genetic differences between two populations are expected to gradually increase as they diverge into two distinct species. Here, we found a gradual increase in genetic differentiation, calculated as *F*_ST_, characterising the transition from populations to species and from the least differentiated intraspecific population pair (Taxon1 OCDA–Taxon1 OCLB, *F*_ST_ = 0.0099) to the most differentiated interspecific population pair (Taxon1 CBHE–Taxon5 OCCH, *F*_ST_ = 0.54) (Figure 4a). This *F*_ST_ gradient illustrates different stages of the speciation continuum along which *S. pistillata* taxa and their populations are evolving. Interestingly, all intraspecific populations pairs had *F*_ST_ <0.16 and all interspecific populations pairs had *F*_ST_ >0.19. In a comparative study investigating the propensity of gene flow between several populations and species pairs, Roux et al. (2016) defined a ‘grey zone’ of speciation, when semi‐isolated populations or species have an intermediate level of divergence. They characterise this range with a net synonymous divergence *D*_a_ between 0.5% and 2% and also propose 0.19 < *F*_ST_ <0.56 and 0.0025 < *D*_*XY*_ <0.0075 as the range for the speciation grey zone (De Jode et al., 2023; Roux et al., 2016). According to our *F*_ST_ estimates, all *S. pistillata* taxa pairs fall within the ‘grey zone’ of speciation, while according to our *D*_*XY*_ estimates, almost all taxa pairs are on the species end of the speciation continuum (Table 1). *D*_*XY*_ should be more reliable given the influence of intraspecific genetic variation on *F*_ST_ (Cruickshank & Hahn, 2014), but our *D*_*XY*_ estimates obtained from RAD‐sequencing data might not be entirely comparable to the *D*_*XY*_ estimates in Roux et al. (2016) who used synonymous sites only.

### 4.2 Speciation with gene flow and genomic heterogeneity in migration

Corals have pelagic larvae and with no complete geographic barriers preventing their dispersal, strictly allopatric speciation seems unlikely (Bolnick & Fitzpatrick, 2007; Feder et al., 2012; Potkamp & Fransen, 2019; Schluter, 2009). In this study, we found strong support for divergence with gene flow among *S. pistillata* taxa, implying that introgression has occurred at some point during their divergence history (Table 1, Table S4). These results are aligned with suspicions of gene flow between coral species (Willis et al., 2006), and recent evidence of historical gene flow between other cryptic coral taxa (Cooke et al., 2020; Fifer et al., 2022; Ladner & Palumbi, 2012; Matias et al., 2022; Prada & Hellberg, 2013, 2021; Prata et al., 2022; Rippe et al., 2021). However, in some interspecific population pairs, we could not differentiate among scenarios of ancient gene flow, secondary contact and continuous gene flow (Table S4), which can be explained by reduced statistical power (i.e., too few SNPs) to model more complex divergence histories or unmodelled population size changes (Momigliano et al., 2020). It remains therefore unclear whether partial reproductive barriers among these taxa have evolved as a result of a gradual reduction in gene flow or because of intermittent historical barriers to gene flow and secondary contact. Since the formation of the GBR around 0.6 Mya, multiple cycles of sea level changes have occurred (Pandolfi & Kelley, 2008) and it is probable that marine species have experienced episodes of allopatry and secondary contact (Laakkonen et al., 2021), which might facilitate reproductive isolation between incipient marine species, including corals (e.g. Duranton et al., 2018; Mao et al., 2018; Rougemont & Bernatchez, 2018; Rougeux et al., 2017; Roux et al., 2013; Zhang et al., 2022). Nevertheless, strict allopatric divergence could confidently be rejected, providing empirical evidence that reproductive isolation between coral taxa can manifest despite continuous or episodic gene flow.

A key prediction of divergence with gene flow theory is that genomic regions under exogenous (e.g. adaptation) or endogenous (e.g. genetic incompatibilities) selection should resist gene flow (Feder et al., 2012; Ravinet et al., 2017; Roux et al., 2016; Wu, 2001). Indeed, we found that the model of continuous heterogeneous gene flow fits the data better than continuous homogeneous gene flow for most population pairs. We estimated that between 6% and 12% of the genome had reduced gene flow rates for within taxa comparisons, and between 9% and 28% for between taxa comparisons (Figure 4d, Table 1). These ‘islands of divergence’ across the *S. pistillata* taxa genomes are in similar proportion compared to European sea bass populations (35%) (Tine et al., 2014) and anchovy ecotypes (20%–25%) (Le Moan et al., 2016), but in smaller proportion compared to *Littorina* sea snails species (67%–77%) (Stankowski et al., 2020). In addition, we found that as populations and taxa diverge, this proportion increases, indicating that more of the genome becomes impermeable to gene exchange. Moreover, similar numbers of highly differentiated and fixed loci were found in sympatry and allopatry, implying that *S. pistillata* taxa are sufficiently reproductively isolated to maintain strong divergence in sympatry in these genomic regions putatively involved in local adaptation or reproductive isolation. However, it is important to note that these patterns of heterogeneous genomic differentiation could also be the result of positive and/or background selection, causing local reductions in genetic diversity (Cruickshank & Hahn, 2014). Further demographic modelling including heterogeneous effective population size across loci could help resolve the relative contributions of the loci associated with reproductive isolation or local adaptation (reduced gene flow) versus the loci impacted by selective sweeps in generating the observed heterogeneous genomic landscapes of divergence.

### 4.3 Rapid evolution of reproductive barriers

In this study, we estimated relatively small gene flow rates among *S. pistillata* taxa (between 0.005 and 0.35 migrant individuals per generation) and we found no evidence of large‐scale recent hybridisation among them. This suggests that present‐day interbreeding is negligible, and that semi‐permeable reproductive barriers among *S. pistillata* taxa evolved in less than 1 million years (estimated split times between 0.42 and 0.92 Mya). This may appear rapid in the marine environment, as there are several examples of longer‐diverged benthopelagic invertebrate species that still hybridise (e.g. *Ciona intestinalis* ascidians, Nydam & Harrison, 2011; *Mytilus* mussels, Bierne et al., 2003). We hypothesise that the brooding life history of *S. pistillata* could explain this rapid diversification. Brooding corals are characterised by reduced planktonic larval duration and local recruitment (Ayre & Hughes, 2000; Underwood et al., 2020), and reduced dispersal has been correlated with greater diversification rates in many organisms (Kisel & Barraclough, 2010; Riginos et al., 2014). It is therefore possible that restricted gene flow due to low dispersal has played an important role in the early stages of speciation among *S. pistillata* taxa. Given their wide and overlapping geographic distributions in the present day, we expect intrinsic pre‐zygotic and/or post‐zygotic reproductive barriers to be in place, such as different spawning times, gamete incompatibilities or high hybrid mortality rates (e.g. Levitan et al., 2004). Our findings contrast to an earlier report that found evidence of hybridisation between *S. pistillata* and *P. damicornis* (Miller & Ayre, 2004), possibly separated by 55 million years (Johnston et al., 2017). We hypothesise that the small sample size and only eight loci screened in that study likely limited their ability to distinguish between incomplete lineage sorting and hybridisation.

In summary, we uncovered that *S. pistillata* from the GBR represents at least five distinct taxa, with reproductive isolation evidenced by sympatric distributions, genome‐wide divergence, Pleistocene divergence times, no definitive hybrid individuals and little ongoing gene exchange.

### 4.4 Niche differences between *Stylophora* sympatric sister taxa

Ecological niche differentiation is the primary mechanism involved in sympatric speciation, allowing the divergence between two taxa that occur within dispersal distance (Faria et al., 2021). Steep environmental gradients characterise coral reefs and produce contrasting habitats at spatial scales allowing gamete mixing (Graham & Nash, 2013). Differentiation by environment is central to ecological speciation and could contribute to the high diversity of corals. We therefore tested whether sympatric *S. pistillata* taxa occupy different environmental niches.

Consistent with niche differentiation, we found strong correlations between *S. pistillata* genetic divergence and specific environmental variables (Figure 5). Specifically, we found that temperature, water clarity, light intensity, current speed and depth explained a significant amount of the genetic divergence between taxa. All these variables have been demonstrated to shape species distributions and coral community assemblages and to affect coral physiology and growth (e.g. Cresswell et al., 2020), with depth often found to partition coral cryptic taxa (e.g. Bongaerts et al., 2013, 2017; Prada & Hellberg, 2021; Prata et al., 2022). Although these RDA results suggest that sympatric *S. pistillata* taxa occupy different environmental niches, investigations of relative abundances by taxon at each site at the reef level did not show any strong patterns of species distribution across small spatial scales (Figure S10). This might be the result of insufficient sample sizes for each taxon at the reef level.

Importantly, as the predominant taxon found in our northernmost sites, Taxon2 may be better adapted to warmer waters than other *S. pistillata* taxa. Differences in heat tolerance have also been evidenced between *Acropora hyacinthus* cryptic species (Rose et al., 2021) and *Pocillopora* sister species (Burgess et al., 2021), and these findings imply that closely related coral taxa might have different resilience levels facing episodic heat waves and continual sea water warming. Further investigations should focus on the physiological differences in these taxa, especially in relation to temperature changes, to better predict their evolutionary trajectories facing climate change.

It is important to note that present‐day environmental conditions do not represent the conditions that taxa experienced during their initial divergence, around 0.5 Mya. We therefore cannot say whether these particular environmental variables have played a role in the initiation of speciation. An alternative speciation scenario that we did not explore is speciation by symbiosis (Brucker & Bordenstein, 2012). One study has found that *S. pistillata* from the Red Sea might be able to vertically transmit their symbionts (Byler et al., 2013), which could extend the coral host heritable genetic variation. Future studies could examine whether different *S. pistillata* taxa harbour different symbionts to explore the possibility of host divergence driven by symbiont co‐adaptation. In this case, environmental association might be secondary but still likely to contribute to the maintenance of genetic distinctiveness among taxa that now occupy wide and overlapping geographic distributions.

### 4.5 Implications for coral taxonomy and conservation

Named species are often the fundamental unit for conservation. In this study, we showed that genomic data can be used to differentiate between closely related taxa where classical taxonomy based on morphology often fails to capture these divergences (Bongaerts et al., 2021). Specifically, we provided multiple lines of evidence for five taxa within *S. pistillata*: multi‐locus genetic divergence, sympatric occurrence at multiple reefs and low gene flow among them and no evidence of recent hybridisation. In addition, we found post hoc differences in gross morphology, with Taxon3 being more compact and harbouring more pronounced corallite hoods and Taxon4 and Taxon5 having thinner and longer branches (Figure S11). To visualise these morphological differences, we used an interactive PCA, which is available here: http://www.cynthiariginos.org/Stylophora.html. Interestingly, Taxon4 and Taxon5 colonies matched the species description of *Stylophora subseriata* (Ehrenberg, 1834), which has never been described on the GBR (Veron & Stafford‐Smith, 2000), and Taxon3 colonies matched the original species description of species *Stylophora mordax* (Dana, 1846), a species that is currently a synonym of *S. pistillata* (Veron & Pichon, n.d.). Taxonomic revisions are outside the scope of this study, and further investigations should examine fine‐scale morphological characters to assign new names and/or resurrect synonymous species of *S. pistillata*. Obtaining genomic data of individuals from other *Stylophora* species might also help place our results in the context of the genus‐wide evolutionary history.

By uncovering the extent of diversity within the *S. pistillata* complex on the GBR, we add to the growing body of literature suggesting that coral diversity is higher than previously thought (Bongaerts et al., 2017; Gijsbers et al., 2023; Matias et al., 2022; Prata et al., 2022; Rippe et al., 2021; Rose et al., 2018; Sturm et al., 2020; Underwood et al., 2020). Coral reef ecosystems, such as the GBR, are rapidly declining due to climate change (Hoegh‐Guldberg et al., 2017), and we need to better understand the extent of coral diversity in order to best protect it (Bickford et al., 2007). Spatial planning and conservation policies often rely on estimates of species richness, population sizes and species ranges, which are inaccurate if multiple taxa are lumped into one taxonomically accepted species. In the case of *S. pistillata*, our findings increase the species richness of most reefs across the GBR, as two or more *S. pistillata* taxa often co‐occur. Our finding of Taxon2 being restricted to the Torres Strait might also warrant special protection although further sampling in the Coral Triangle is needed. Species lumping inevitably inflates population sizes estimates, and extinction risk predictions based these estimates (e.g. Dietzel et al., 2021) therefore need to be revised. In addition, our results are consistent with divergent lineages being differentially adapted to their environment and it is therefore possible that they follow different evolutionary trajectories in response to ongoing climate change. We could, for example, hypothesise that *S. pistillata* Taxon2 might be better adapted to cope with higher temperatures.

Fundamental knowledge regarding species identity is also important in the context of reef restoration. As restoration efforts such as assisted gene flow and genetic rescue are being considered for corals (Quigley et al., 2019; van Oppen et al., 2017), a better understanding of what constitutes cohesive coral taxa is critical. In particular, the movement of recruits or larvae will not help replenish stock or facilitate adaptation if they cannot interbreed with the targeted population. For example, Torres Strait coral populations might be chosen as a source population to introduce heat‐resistant alleles into more vulnerable GBR populations but in the case of *S. pistillata*, our results suggest that possible reproductive isolation between Taxon2 in the Torres Strait and any taxon from the GBR will impede these interventions.

## CONFLICT OF INTEREST STATEMENT

The authors have no conflict of interest to declare.

## Supporting information


Figure S1.

Table S1.

## Data Availability

Raw sequence reads for this study are available on NCBI (BioProject PRJNA996644) and all sample metadata including photographs of genotyped individuals can be accessed on GEOME (https://geome‐db.org/record/ark:/21547/FLu2). To visualise morphological differences among colonies, an interactive PCA is available online at https://riginosresearch.github.io/stylophora_interactive_pca.html. Unfiltered VCF file and scripts are available on GitHub (https://github.com/zoemeziere/Stylophora_speciation).
