# Supplementary material for: Exploring coral speciation: Multiple sympatric Stylophora pistillata taxa along a divergence continuum on the Great Barrier Reef
Source: Evol Appl. 2024 Jan 26;17(1):e13644. doi: 10.1111/eva.13644 (PMC10818133; doi:10.1111/eva.13644)
Supplement: Supplementary file 1 — Figure S1. [file EVA-17-e13644-s001.zip › evs13644-sup-0001-Figures.pdf]

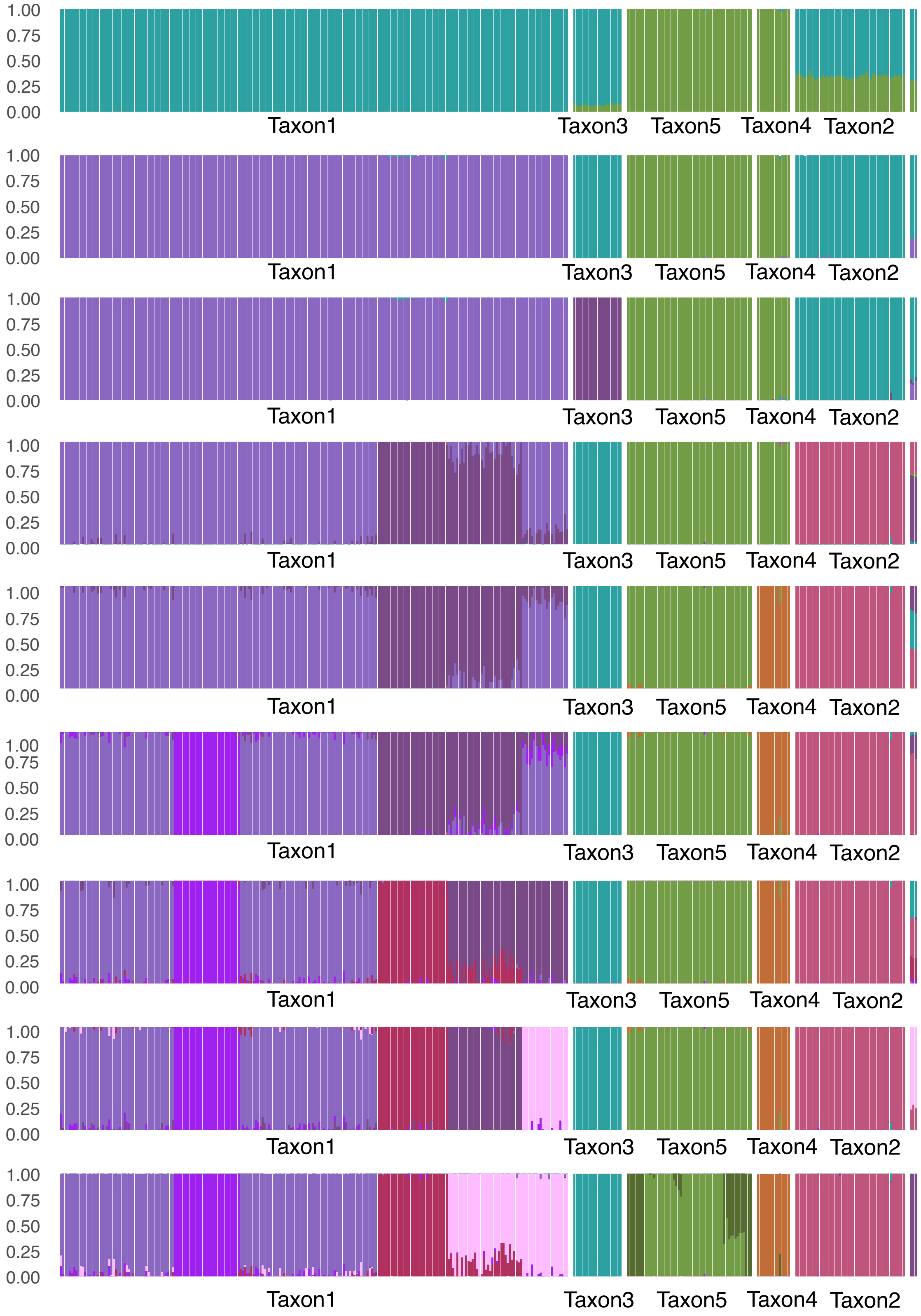
1.00
0.75
0.50
0.25
0.00
Taxon1
Taxon3
Taxon5
Taxon4
Taxon2

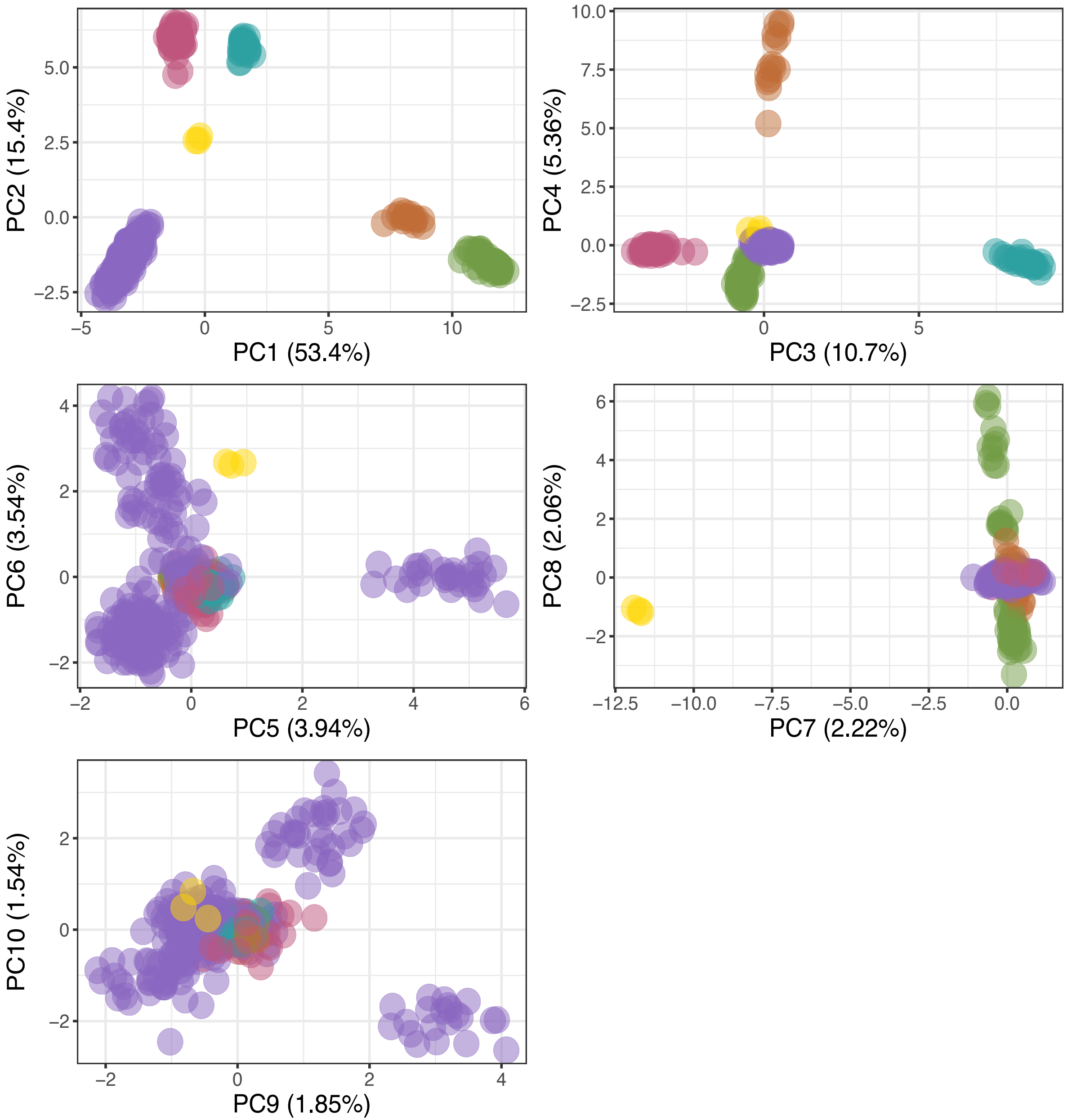

PC1 (53.4%)
PC2 (15.4%)
PC3 (10.7%)
PC4 (5.36%)
PC5 (3.94%)
PC6 (3.54%)
PC7 (2.22%)
PC8 (2.06%)
PC9 (1.85%)
PC10 (1.54%)

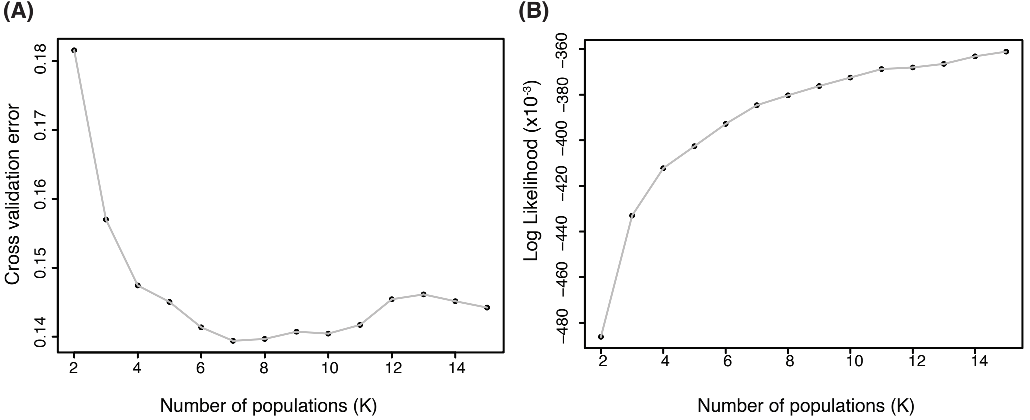

(A)
Cross validation error
0.14
0.15
0.16
0.17
0.18
2
4
6
8
10
12
14
Number of populations (K)
(B)
Log Likelihood (x10^{-3})
−480
−460
−440
−420
−400
−380
−360
2
4
6
8
10
12
14
Number of populations (K)

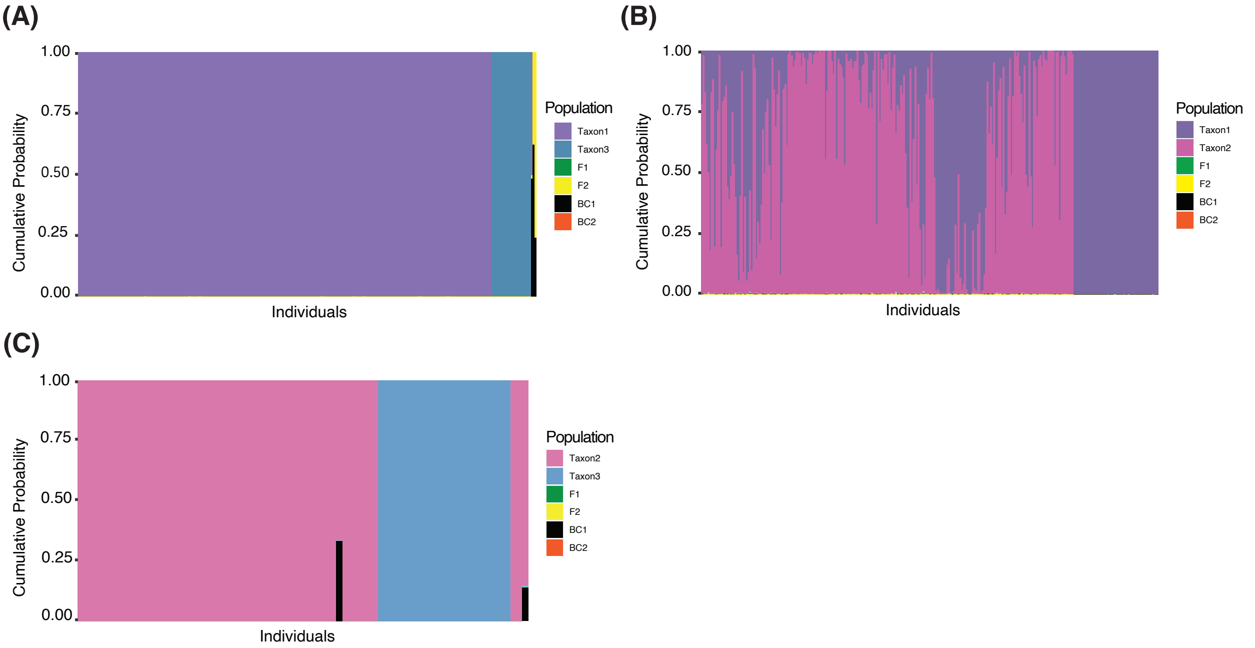

(A)
Cumulative Probability
1.00
0.75
0.50
0.25
0.00
Individuals
Population
Taxon1
Taxon3
F1
F2
BC1
BC2
(B)
Cumulative Probability
1.00
0.75
0.50
0.25
0.00
Individuals
Population
Taxon1
Taxon2
F1
F2
BC1
BC2
(C)
Cumulative Probability
1.00
0.75
0.50
0.25
0.00
Individuals
Population
Taxon2
Taxon3
F1
F2
BC1
BC2

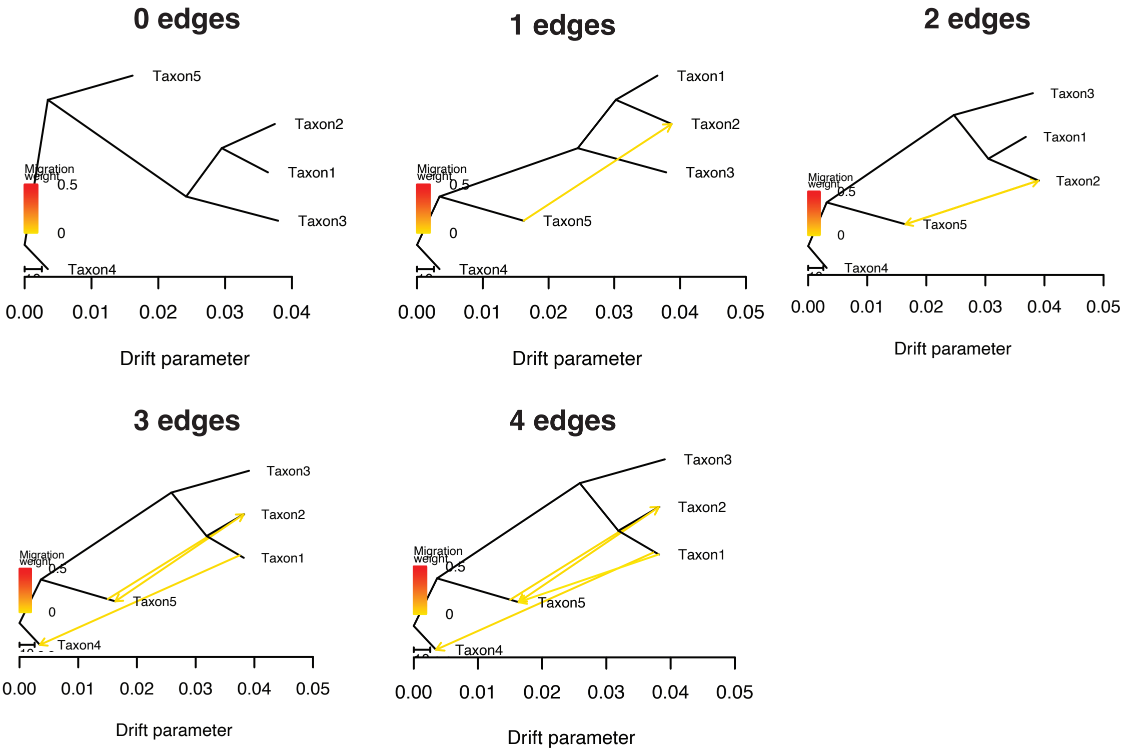
0 edges
Taxon5
Taxon2
Taxon1
Taxon3
Taxon4
Migration weight
0.5
0
0.00
0.01
0.02
0.03
0.04
Drift parameter
1 edges
Taxon1
Taxon2
Taxon3
Taxon5
Taxon4
Migration weight
0.5
0
0.00
0.01
0.02
0.03
0.04
0.05
Drift parameter
2 edges
Taxon3
Taxon1
Taxon2
Taxon5
Taxon4
Migration weight
0.5
0
0.00
0.01
0.02
0.03
0.04
0.05
Drift parameter
3 edges
Taxon3
Taxon2
Taxon1
Taxon5
Taxon4
Migration weight
0.5
0
0.00
0.01
0.02
0.03
0.04
0.05
Drift parameter
4 edges
Taxon3
Taxon2
Taxon1
Taxon5
Taxon4
Migration weight
0.5
0
0.00
0.01
0.02
0.03
0.04
0.05
Drift parameter

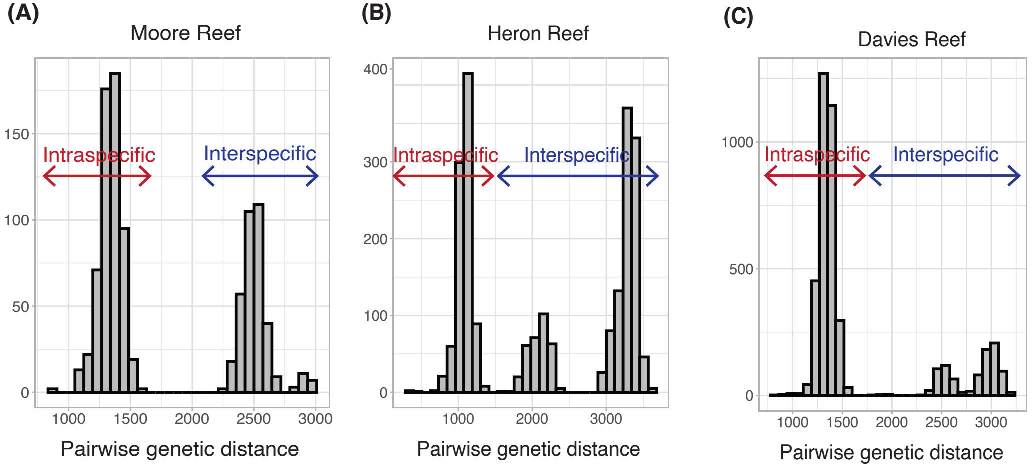
(A)
Moore Reef
Intraspecific
Interspecific
0
50
100
150
1000
1500
2000
2500
3000
Pairwise genetic distance
(B)
Heron Reef
Intraspecific
Interspecific
0
100
200
300
400
1000
2000
3000
Pairwise genetic distance
(C)
Davies Reef
Intraspecific
Interspecific
0
500
1000
1000
1500
2000
2500
3000
Pairwise genetic distance

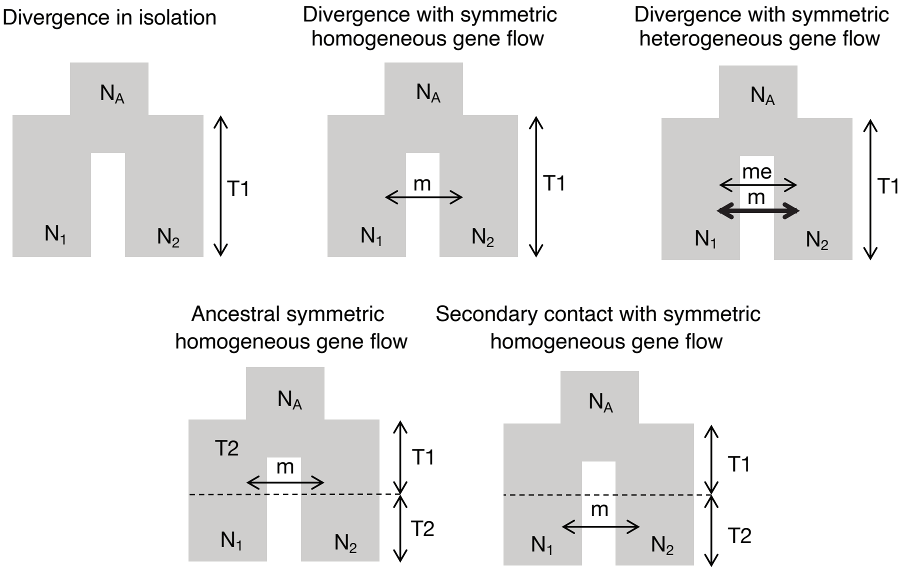
Divergence in isolation
N_A
N_1
N_2
T1
Divergence with symmetric homogeneous gene flow
N_A
m
N_1
N_2
T1
Divergence with symmetric heterogeneous gene flow
N_A
me
m
N_1
N_2
T1
Ancestral symmetric homogeneous gene flow
N_A
T2
m
N_1
N_2
T1
T2
Secondary contact with symmetric homogeneous gene flow
N_A
m
N_1
N_2
T1
T2

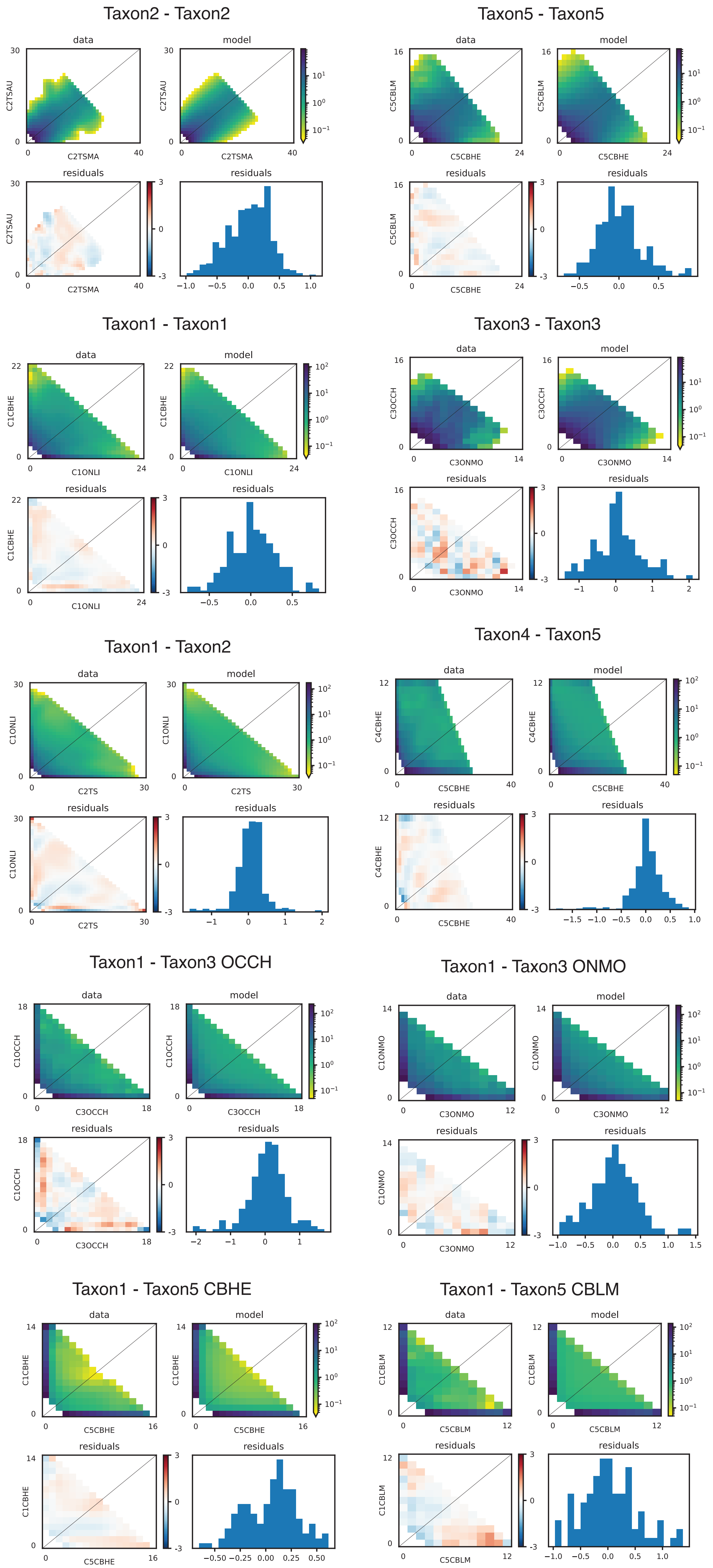

Taxon2 - Taxon2
data
model
residuals
C2TSAU
C2TSMA
Taxon5 - Taxon5
C5CBLM
C5CBHE
Taxon1 - Taxon1
C1CBHE
C1ONLI
Taxon3 - Taxon3
C3OCCH
C3ONMO
Taxon1 - Taxon2
C1ONLI
C2TS
Taxon4 - Taxon5
C4CBHE
C5CBHE
Taxon1 - Taxon3 OCCH
C1OCCH
C3OCCH
Taxon1 - Taxon3 ONMO
C1ONMO
C3ONMO
Taxon1 - Taxon5 CBHE
C1CBHE
C5CBHE
Taxon1 - Taxon5 CBLM
C1CBLM
C5CBLM

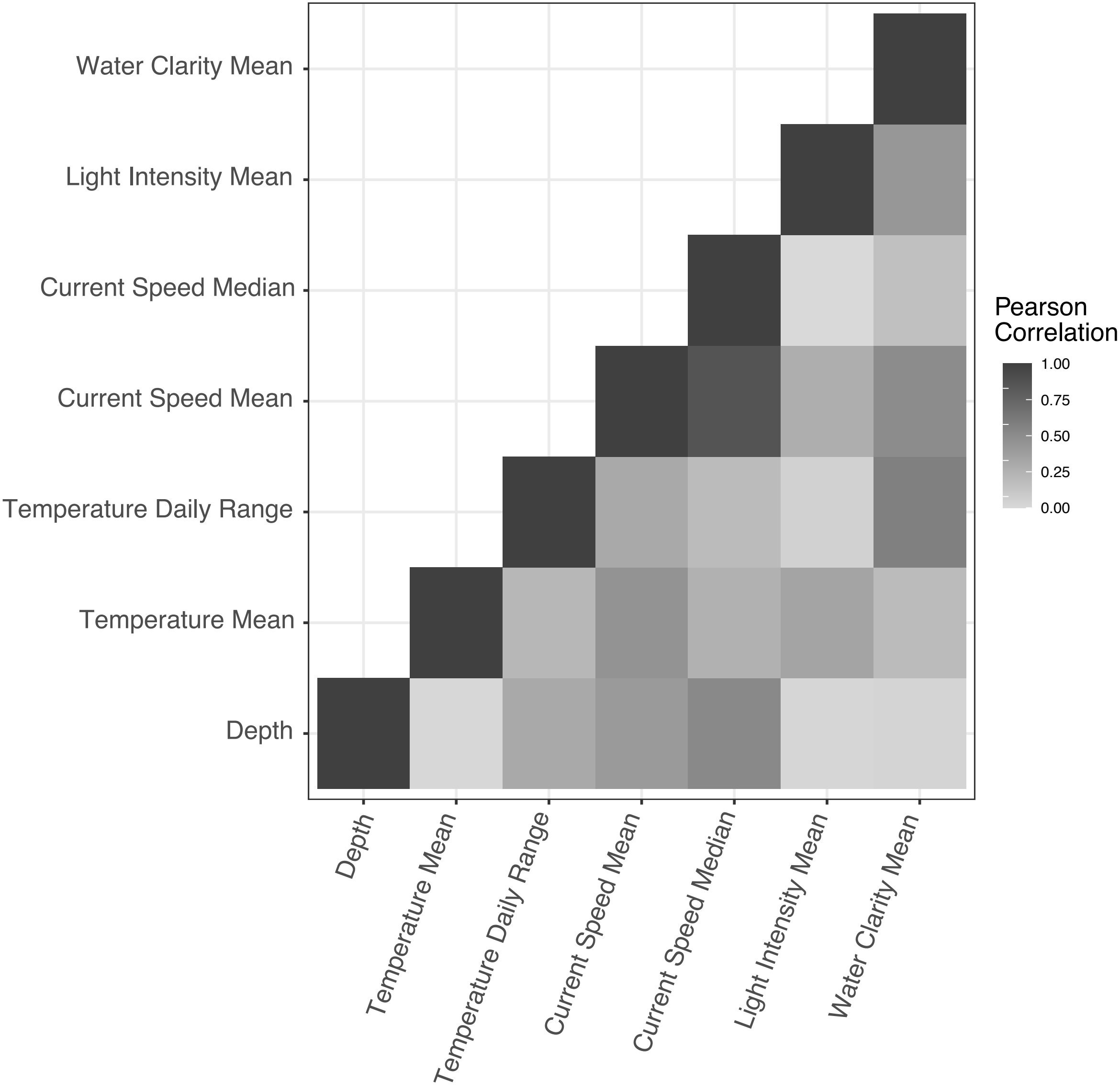
Water Clarity Mean
Light Intensity Mean
Current Speed Median
Current Speed Mean
Temperature Daily Range
Temperature Mean
Depth
Depth
Temperature Mean
Temperature Daily Range
Current Speed Mean
Current Speed Median
Light Intensity Mean
Water Clarity Mean
Pearson Correlation
1.00
0.75
0.50
0.25
0.00

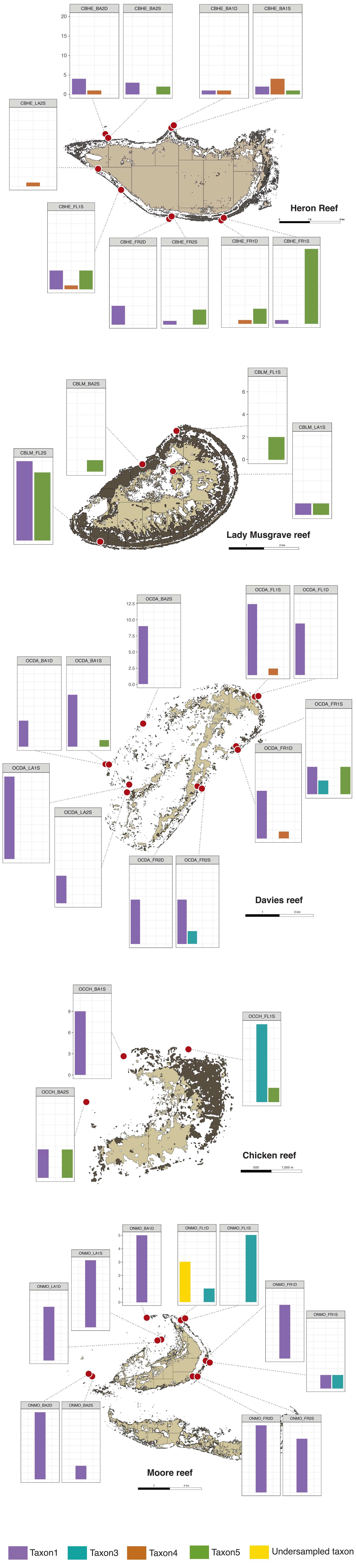
CBHE_BA2D
CBHE_BA2S
CBHE_BA1D
CBHE_BA1S
20
15
10
5
0
CBHE_LA2S
CBHE_FL1S
Heron Reef
CBHE_FR2D
CBHE_FR2S
CBHE_FR1D
CBHE_FR1S
CBLM_BA2S
CBLM_FL1S
6
4
2
0
CBLM_LA1S
CBLM_FL2S
Lady Musgrave reef
OCDA_BA2S
12.5
10.0
7.5
5.0
2.5
0.0
OCDA_FL1S
OCDA_FL1D
OCDA_BA1D
OCDA_BA1S
OCDA_FR1S
OCDA_LA1S
OCDA_FR1D
OCDA_LA2S
OCDA_FR2D
OCDA_FR2S
Davies reef
OCCH_BA1S
9
6
3
0
OCCH_FL1S
OCCH_BA2S
Chicken reef
500
1,000 m
ONMO_BA1D
ONMO_FL1D
ONMO_FL1S
5
4
3
2
1
0
ONMO_LA1S
ONMO_LA1D
ONMO_FR1D
ONMO_FR1S
ONMO_BA2D
ONMO_BA2S
ONMO_FR2D
ONMO_FR2S
Moore reef
Taxon1
Taxon3
Taxon4
Taxon5
Undersampled taxon

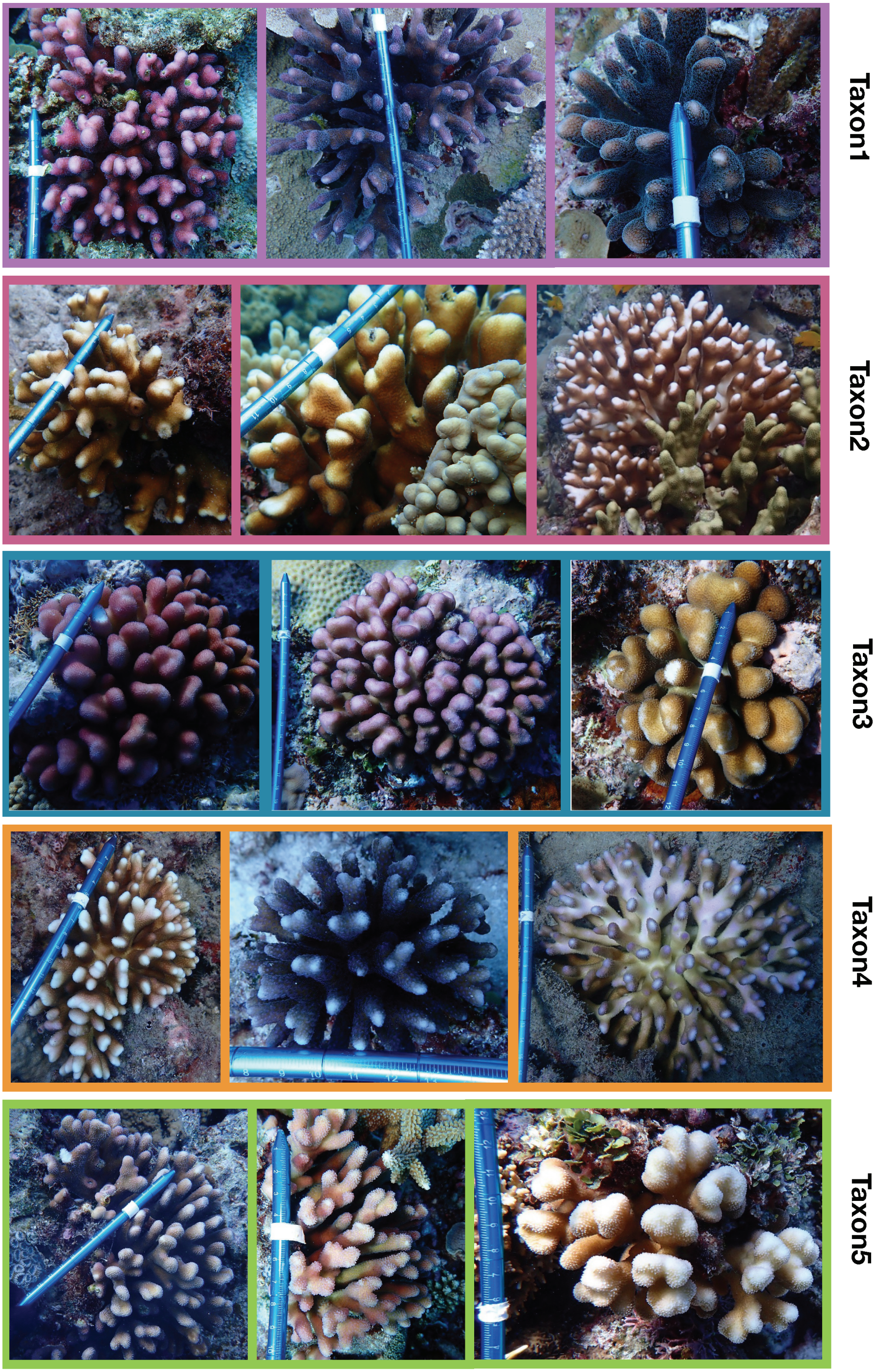
Taxon1
Taxon2
Taxon3
Taxon4
Taxon5
