# Supplementary material for: Exploring coral speciation: Multiple sympatric Stylophora pistillata taxa along a divergence continuum on the Great Barrier Reef
Source: Evol Appl. 2024 Jan 26;17(1):e13644. doi: 10.1111/eva.13644 (PMC10818133; doi:10.1111/eva.13644)
Supplement: Supplementary file 1 — Figure S1. [file EVA-17-e13644-s001.zip › SuppFiguresLegends_1123.docx]

**Supplementary figures**

**Supplementary Figure 1**: *Stylophora pistillata* population genetic structure inferred from ADMIXTURE analyses with K=2 to K=10. Individuals are represented as vertical stacked bars, indicating the proportion of ancestry from the K constructed ancestral populations.

**Supplementary Figure 2**: *Stylophora pistillata* population genetic structure represented by the first ten axes of the Principal Component Analysis. Individuals are represented as dotes, coloured by taxon.

**Supplementary Figure 3**: (A) Cross Validation errors and (B) Log likelihoods obtained from ADMIXURE runs with K=2 to K=15.

**Supplementary Figure 4**: NewHybrids analyses show that the three putative hybrid individuals are not definite early generation hybrids between *Stylophora pistillata* Taxon1, Taxon2 and Taxon3. Each line corresponds to the posterior probability of assignment to either parental population or to a hybrid class. The last three individuals are the putative hybrids.

**Supplementary Figure 5**: Treemix inferred phylogeny with zero to four migration edges between all five *Stylophora pistillata* taxa.

**Supplementary Figure 6**: Pairwise Euclidean genetic distance frequency distribution between all *Stylophora pistillata* individuals from (A) Moore reef, (B) Heron reef and (C) Davies reef reveal multiple peaks of genetic distances representing intraspecific genetic distances (~ <1700 allelic differences) and interspecific genetic distances (~ >1700 allelic differences) and suggesting the existence of multiple sympatric species at these reefs.

**Supplementary Figure 7**: Schematics of five models used in the dadi demographic analyses and specific parameters estimated: N1 and N2 are the population sizes of each population after the split, m is the symmetric gene flow rate between populations, me is the symmetric reduced gene flow rate between populations, T1 is the time between the split and present or T2 and T2 is the time between T1 and present.

**Supplementary Figure 8**: Data Folded Joint Allele Frequency Spectra, simulated Joint Allele Frequency Spectra of the most likely dadi demographic model (divergence with symmetric heterogeneous gene flow) and the standardised residuals for the model against the data, for all population pairs.

**Supplementary Figure 9:** Correlation coefficients among the seven environmental variables considered for the explanatory matrix in the RDA analysis.

**Supplementary Figure 10**: Site-level spatial distribution of *Stylophora pistillata* taxa on reefs where more than one taxon was sampled. Base maps obtained via the Allen coral atlas (https://allencoralatlas.org/), showing reefs in brown and sand in beige.

**Supplementary Figure 11**: Photographs showing the gross morphology of selected individuals from each *Stylophora pistillata* genetically distinct taxa
