# Supplementary material for: Exploring coral speciation: Multiple sympatric Stylophora pistillata taxa along a divergence continuum on the Great Barrier Reef
Source: Evol Appl. 2024 Jan 26;17(1):e13644. doi: 10.1111/eva.13644 (PMC10818133; doi:10.1111/eva.13644)
Supplement: Supplementary file 2 — Table S1. [file EVA-17-e13644-s002.docx]

**Supplementary Table 1**: Details on the sampling sites where the 379 genotyped *Stylophora pistillata* individuals were collected.

| Reef | Site | Habitat | Depth | Latitude | Longitude | Number of samples |
| --- | --- | --- | --- | --- | --- | --- |
| Davies | OCDA_BA1D | Back | Deep | -18.8258 | 147.6274 | 4 |
| Davies | OCDA_BA1S | Back | Shallow | -18.8259 | 147.6273 | 9 |
| Davies | OCDA_BA2S | Back | Shallow | -18.8145 | 147.6372 | 9 |
| Davies | OCDA_FL1D | Flank | Deep | -18.8059 | 147.6688 | 8 |
| Davies | OCDA_FL1S | Flank | Shallow | -18.8058 | 147.6686 | 12 |
| Davies | OCDA_FR1D | Front | Deep | -18.8197 | 147.6639 | 8 |
| Davies | OCDA_FR1S | Front | Shallow | -18.8192 | 147.6639 | 10 |
| Davies | OCDA_FR2D | Front | Deep | -18.8319 | 147.6526 | 7 |
| Davies | OCDA_FR2S | Front | Shallow | -18.8319 | 147.6525 | 9 |
| Davies | OCDA_LA1S | Lagoon | Shallow | -18.833 | 147.6334 | 12 |
| Davies | OCDA_LA2S | Lagoon | Shallow | -18.8313 | 147.6332 | 4 |
| Pelorus | PAOR_FL1D | Flank | Deep | -18.5715 | 146.4954 | 6 |
| Pelorus | PAPE_BA1D | Back | Deep | -18.5505 | 146.4882 | 3 |
| Pelorus | PAPE_BA1S | Back | Shallow | -18.5508 | 146.4884 | 1 |
| Pelorus | PAPE_BA2D | Back | Deep | -18.5408 | 146.4885 | 3 |
| Pelorus | PAPE_BA2S | Back | Shallow | -18.541 | 146.4886 | 3 |
| Pelorus | PAPE_FR1D | Front | Deep | -18.5394 | 146.4995 | 10 |
| Pelorus | PAPE_FR1S | Front | Shallow | -18.5395 | 146.4992 | 1 |
| Aukane | TSAU_BA1S | Back | Shallow | -9.86762 | 143.3894 | 3 |
| Aukane | TSAU_BA2S | Back | Shallow | -9.8757 | 143.3885 | 5 |
| Aukane | TSAU_FL1S | Flank | Shallow | -9.88203 | 143.3941 | 5 |
| Aukane | TSAU_FR1S | Front | Shallow | -9.87996 | 143.41 | 6 |
| Aukane | TSAU_FR2S | Front | Shallow | -9.88359 | 143.4071 | 1 |
| Dungeness | TSDU_BA3S | Back | Shallow | -9.98532 | 142.9085 | 2 |
| Dungeness | TSDU_FR1S | Back | Shallow | -9.9312 | 143.0054 | 1 |
| Dungeness | TSDU_FR2S | Front | Shallow | -10.0371 | 142.9265 | 1 |
| Masig | TSMA_BA1S | Back | Shallow | -9.7489 | 143.3998 | 3 |
| Masig | TSMA_BA2D | Back | Deep | -9.74409 | 143.4157 | 3 |
| Masig | TSMA_BA2S | Back | Shallow | -9.74424 | 143.4158 | 4 |
| Masig | TSMA_FL1S | Flank | Shallow | -9.7599 | 143.3980 | 2 |
| Masig | TSMA_FR1S | Front | Shallow | -9.76224 | 143.4214 | 4 |
| Masig | TSMA_FR2D | Front | Deep | -9.74637 | 143.4601 | 1 |
| Masig | TSMA_FR2S | Front | Shallow | -9.74838 | 143.4585 | 5 |
| Heron | CBHE_BA1D | Back | Deep | -23.428 | 151.9525 | 2 |
| Heron | CBHE_BA1S | Back | Shallow | -23.4284 | 151.9524 | 7 |
| Heron | CBHE_BA2D | Back | Deep | -23.4341 | 151.9203 | 5 |
| Heron | CBHE_BA2S | Back | Shallow | -23.434 | 151.9209 | 5 |
| Heron | CBHE_FL1S | Flank | Shallow | -23.4562 | 151.9257 | 11 |
| Heron | CBHE_FR1D | Front | Deep | -23.4721 | 151.9779 | 5 |
| Heron | CBHE_FR1S | Front | Shallow | -23.472 | 151.9777 | 21 |
| Heron | CBHE_FR2D | Front | Deep | -23.4708 | 151.9511 | 5 |
| Heron | CBHE_FR2S | Front | Shallow | -23.4704 | 151.9507 | 5 |
| Heron | CBHE_LA1S | Lagoon | Shallow | -23.446 | 151.9147 | 1 |
| Lady Musgrave | CBLM_BA2S | Back | Shallow | -23.8943 | 152.4044 | 1 |
| Lady Musgrave | CBLM_FL1S | Flank | Shallow | -23.8849 | 152.4136 | 2 |
| Lady Musgrave | CBLM_FL2S | Flank | Shallow | -23.9176 | 152.3920 | 13 |
| Lady Musgrave | CBLM_LA1S | Lagoon | Shallow | -23.8961 | 152.4138 | 2 |
| Chicken | OCCH_BA1S | Back | Shallow | -18.6532 | 147.7085 | 9 |
| Chicken | OCCH_BA2S | Back | Shallow | -18.6604 | 147.7027 | 4 |
| Chicken | OCCH_FL1S | Flank | Shallow | -18.6522 | 147.7185 | 13 |
| Little Broadhurst | OCLB_BA1S | Back | Shallow | -18.9493 | 147.6929 | 8 |
| Little Broadhurst | OCLB_FL1S | Flank | Shallow | -18.9441 | 147.6993 | 7 |
| Little Broadhurst | OCLB_LA1S | Lagoon | Shallow | -18.9494 | 147.7019 | 6 |
| Lizard | ONLI_BA1S | Back | Shallow | -14.6507 | 145.4501 | 3 |
| Lizard | ONLI_BA2S | Back | Shallow | -14.6677 | 145.4418 | 6 |
| Lizard | ONLI_FL1S | Flank | Shallow | -14.6980 | 145.4454 | 3 |
| Lizard | ONLI_FR1S | Front | Shallow | -15.7552 | 145.7388 | 8 |
| Lizard | ONLI_FR2S | Front | Shallow | -14.6494 | 145.4929 | 7 |
| Lizard | ONLI_LA1S | Lagoon | Shallow | -14.6877 | 145.4652 | 5 |
| Moore | ONMO_BA1D | Back | Deep | -16.8471 | 146.2174 | 5 |
| Moore | ONMO_BA2D | Back | Deep | -16.8819 | 146.1837 | 5 |
| Moore | ONMO_BA2S | Back | Shallow | -16.8820 | 146.1839 | 1 |
| Moore | ONMO_FL1D | Flank | Deep | -16.8484 | 146.2372 | 4 |
| Moore | ONMO_FL1S | Flank | Shallow | -16.8485 | 146.2372 | 5 |
| Moore | ONMO_FR1D | Front | Deep | -16.8720 | 146.2536 | 4 |
| Moore | ONMO_FR1S | Front | Shallow | -16.8720 | 146.2536 | 2 |
| Moore | ONMO_FR2D | Front | Deep | -16.8830 | 146.2448 | 5 |
| Moore | ONMO_FR2S | Front | Shallow | -16.8826 | 146.2446 | 4 |
| Moore | ONMO_LA1S | Lagoon | Shallow | -16.85984 | 146.2256 | 4 |
| Moore | ONMO_LA1D | Lagoon | Deep | -16.85992 | 146.2257 | 5 |

**Supplementary Table 2**: Description and acquisition details on the environmental variables used as explanatory variables for the partial RDA.

| Variable | Description | Unit | Depths | Source | Resolution | Summary type | Time step | Date range |
| --- | --- | --- | --- | --- | --- | --- | --- | --- |
| Current speed mean | Euclidean distance of eastward and northward currents | m•s^-1^ | 5m and 13m | eReefs hydrodynamic models | 1km | Mean | Daily | 01/01/2014 to 06/05/2021 |
| Current speed median | Euclidean distance of eastward and northward currents | m•s^-1^ | 5m and 13m | eReefs hydrodynamic models | 1km | Median | Daily | 01/01/2014 to 06/05/2021 |
| Temperature mean | Temperature | degrees Celsius | 5m and 13m | eReefs hydrodynamic models | 1km | Mean | Daily | 01/01/2014 to 06/05/2021 |
| Temperature median | Temperature | degrees Celsius | 5m and 13m | eReefs hydrodynamic models | 1km | Median | Daily | 01/01/2014 to 06/05/2021 |
| Temperature max | Temperature | degrees Celsius | 5m and 13m | eReefs hydrodynamic models | 1km | Maximum | Daily | 01/01/2014 to 06/05/2021 |
| Temperature daily range | Temperature | degrees Celsius | 5m and 13m | eReefs hydrodynamic models | 1km | Daily range | Daily | 01/01/2014 to 06/05/2021 |
| Light intensity mean | Light intensity above seagrass (PAR) | mol•photon•m-2•d^-1^ | 5.5m and 12.75m | eReefs biogeochemical models | 4km | Mean | Daily | 01/12/2010 to 01/12/2018 |
| Light intensity median | Light intensity above seagrass (PAR) | mol•photon•m-2•d^-1^ | 5.5m and 12.75m | eReefs biogeochemical models | 4km | Median | Daily | 01/12/2010 to 01/12/2018 |
| Light intensity max | Light intensity above seagrass (PAR) | mol•photon•m-2•d^-1^ | 5.5m and 12.75m | eReefs biogeochemical models | 4km | Maximum | Daily | 01/12/2010 to 01/12/2018 |
| Water clarity mean | Logarithm of the vertical attenuation coefficient of light at 488m scaled by depth | meters | 5.5m and 12.75m | eReefs biogeochemical models | 4km | Mean | Daily | 01/12/2010 to 01/12/2018 |
| Water clarity median | Logarithm of the vertical attenuation coefficient of light at 488m scaled by depth | meters | 5.5m and 12.75m | eReefs biogeochemical models | 4km | Median | Daily | 01/12/2010 to 01/12/2018 |
| Water clarity max | Logarithm of the vertical attenuation coefficient of light at 488nm scaled by depth | meters | 5.5m and 12.75m | eReefs biogeochemical models | 4km | Maximum | Daily | 01/12/2010 to 01/12/2018 |
| Depth | Bottom depth | meters |  | Field | 0.1m | Maximum |  |  |
|  |  |  |  |  |  |  |  |  |

|  |  |  |  | | Demographic modelling results | | | | | | | | |  |
| --- | --- | --- | --- | --- | --- | --- | --- | --- | --- | --- | --- | --- | --- | --- |
| Level | **Taxa pair** | **Population pair** | **N_ref_** | **𝛎_1_** | | **𝛎_2_** | **m** | **m_e_** | **M_21_** | **M_12_** | **T** | **P** | **Q** | |
| Intraspecific | Taxon2 | T2TSAU-T2TSMA | 4548 ± 476 | 1027  ± 2365 | | 6194 ± 1000 | 1.0e-3  ± 1.3e-4 | 2.5e-4  ± 1.0e-4 | 10.6 ± 1.2 | 6.4 ± 0.12 | 0.037 ± 9.5e-3 | 0.94 ± 0.068 | 0.056 ± 0.068 | |
|  | Taxon5 | T5CBLM-T5CBHE | 47903 ± 11509 | 70878 ± 19640 | | 59012 ± 1964 | 2.7e-5 ± 5.3e-8 | 7.4e-6 ± 7.5e-6 | 1.9 ± 0.26 | 1.6 ± 0.14 | 0.37 ± 0.22 | 0.90 ± 0.037 | 0.10 ± 0.037 | |
|  | Taxon3 | T3OCCH-T3ONMO | 43561 ± 4562 | 81787 ± 40076 | | 128577 ± 40976 | 1.5e-5 ± 4.8e-7 | 1.4e-6 ±  7.4e-7 | 1.3 ±  0.31 | 2.0 ±  0.23 | 0.45 ±  0.26 | 0.94 ±  0.044 | 0.060 ±  0.044 | |
|  | Taxon1 | T1CBHE-T1ONLI | 27679 ± 7527 | 31020 ± 7750 | | 127982 ± 7750 | 1.0e-5 ± 2.8e-6 | 5.2e-6 ± 2.8e-6 | 0.32 ± 0.04 | 1.35 ± 0.083 | 0.38 ± 0.17 | 0.88 ± 0.010 | 0.12 ± 0.10 | |
| Interspecific | Taxon1-Taxon2 | T1ONLI-T2TSAU | 43026 ± 3656 | 134733 ± 14198 | | 156535 ± 14198 | 3.0e-6 ± 1.0e-6 | 1.5e-8 ± 1.1e-9 | 0.41 ± 0.021 | 0.47 ± 1.1e-4 | 0.44 ± 0.057 | 0.91 ± 0.070 | 0.090 ± 0.070 | |
|  | Taxon4-Taxon5 | T4CBHE -T5CBHE | 32228 ± 6503 | 91660 ± 19014 | | 58706 ± 19014 | 4.2e-6 ± 7.9e-7 | 1.5e-8 ± 5.2e-9 | 0.39 ± 0.040 | 0.25 ± 9.2e-5 | 0.42 ± 0.13 | 0.89 ± 0.076 | 0.11 ± 0.076 | |
|  | Taxon1-Taxon3 | T1OCCH-T3OCCH | 26159 ± 3552 | 119046 ± 15975 | | 113869 ± 15957 | 1.3e-6 ± 1.8e-7 | 8.8e-8 ± 1.0e-9 | 0.16 ± 0.010 | 0.15 ± 6.7e-4 | 0.54 ± 0.086 | 0.82 ± 0.061 | 0.18 ± 0.061 | |
|  | Taxon1-Taxon3 | T1ONMO-T3ONMO | 15843 ± 4296 | 143123 ± 38023 | | 148824 ± 38023 | 5.4e-7 ± 1.9e-8 | 5.4e-8 ± 3.1e-9 | 0.078 ± 0.010 | 0.080 ± 1.1e-3 | 0.89 ± 0.26 | 0.78 ± 0.092 | 0.22 ± 0.092 | |
|  | Taxon1-Taxon5 | T1CBLM-T5CBLM | 271224 ± 5456 | 110669 ± 24954 | | 89702 ± 5808 | 7.3e-8 ± 7.4e-8 | 1.8e-8 ± 1.8e-8 | 8.1e-3 ± 9.2e-4 | 6.6e-3 ± 1.6e-4 | 0.66 ± 0.16 | 0.72 ± 0.044 | 0.28 ± 0.044 | |
|  | Taxon1-Taxon5 | T1CBHE-T5CBHE | 8282  ± 4699 | 100038 ± 5808 | | 93345 ± 24954 | 2.4e-7 ± 1.1e-7 | 6.0e-8 ±  6.0e-8 | 0.024 ± 7.1e-3 | 0.022 ± 1.9e-3 | 0.92 ± 0.55 | 0.72 ± 0.025 | 0.28 ± 0.2025 | |

**Supplementary Table 3**: Results from the best demographic model (divergence with heterogeneous symmetric gene flow) performed in dadi on selected populations, including confidence intervals around each inferred parameter. 𝛎_1_ and 𝛎_2_ are the estimated population sizes, in number of individuals; m is the estimated heterogeneous symmetric gene flow rate between populations every generation; m_e_ is the reduced gene flow rate between populations every generation; M_21_ and M_12_ are the estimated number of migrant individuals from Population2 to Population1 and from Population1 to Population2 respectively, every generation; T is the estimated divergence time, in millions of years, P is the proportion of the genome evolving neutrally and Q is the proportion of the genome experiencing reduced gene flow.

**Supplementary Table 4**: Maximum likelihood, AIC and estimated scaled parameters for the best-fit demographic model for each divergence scenario. N_ref_ is the ancestral population size, in number of individuals; 𝛎1 and 𝛎2 are the estimated population sizes, in number of individuals; m is the estimated symmetrical gene flow rate between populations every generation; m_e_ is the reduced gene flow rate between populations every generation, M_21_ and M_12_ are the estimated gene flow rates, in number of migrants from Population2 to Population1 and from Population1 to Population2 respectively, every generation; T1 and T2 are the estimated divergence time, in millions of years, P is the proportion of the genome evolving neutrally. Best fit models are in bold.

| Population pairs | Model | Log likelihood | AIC | N_ref_ | 𝛎1 | 𝛎2 | m | m_e_ | M_21_ | M_12_ | T1 | T2 | P |
| --- | --- | --- | --- | --- | --- | --- | --- | --- | --- | --- | --- | --- | --- |
| T2TSAU-T2TSMA | Isolation | -645.9 | 1297.9 | 73942 | 581454 | 970163 | - | - | - | - | 0.069 | - | - |
|  | Homo. sym. gene flow | -558.2 | 1124.5 | 46388 | 103446 | 44533 | 1.0e-4 | - | 11.1 | 4.8 | 0.25 | - | - |
|  | **Hetero sym. gene flow** | **-550.3** | **1112.7** | **4548** | **10207** | **6194** | **1.0e-3** | **2.5e-4** | **10.6** | **6.4** | **0.03** | **-** | **0.94** |
| T5CBLM-T5CBHE | Isolation | -327.3 | 660.7 | 78998 | 113079 | 1070759 | - | - | - | - | 0.094 | - | - |
|  | **Homo. sym. gene flow** | **-305.8** | **619.7** | **55503** | **72154** | **55503** | **2.1e-5** | **-** | **1.5** | **1.2** | **0.29** | **-** | **-** |
|  | **Hetero sym. gene flow** | **-303.7** | **619.4** | **47093** | **70878** | **59012** | **2.7e-5** | **7.4e-6** | **1.9** | **1.6** | **0.37** | **-** | **0.90** |
| T3OCCH-T3ONMO | Isolation | -271.1 | 548.3 | 84189 | 732726 | 457940 | - | - | - | - | 0.23 | - | - |
|  | **Homo. sym. gene flow** | **-223.9** | **455.8** | **43523** | **81775** | **128502** | **1.5e-5** | **-** | **1.3** | **2.0** | **0.24** | **-** | **-** |
|  | **Hetero sym. gene flow** | **-223.9** | **459.8** | **43561** | **81787** | **128577** | **1.5e-5** | **1.4e-5** | **1.3** | **2.0** | **0.45** | **-** | **0.94** |
| T1CBHE-T1ONLI | Isolation | -449.1 | 904.3 | 67532 | 32557 | 195790 | - | - | - | - | 0.056 | - | - |
|  | Homo. sym. gene flow | -382.6 | 773.3 | 31414 | 30770 | 127989 | 1.2e-5 | - | 0.3 | 1.5 | 0.36 | - | - |
|  | **Hetero sym. gene flow** | **-379.7** | **771.4** | **27679** | **31020** | **127982** | **1.0e-5** | **5.1e-6** | **0.3** | **1.3** | **0.38** | **-** | **0.88** |
| T1ONLI-T2TS | Isolation | -753.1 | 1512.3 | 87579 | 197571 | 196301 | - | - | - | - | 0.21 | - | - |
|  | Homo. sym. gene flow | -557.6 | 1123.3 | 39681 | 132252 | 151486 | 2.7e-6 | - | 0.3 | 0.41 | 0.47 | - | - |
|  | **Hetero sym. gene flow** | **-553.4** | **1118.9** | **43026** | **134733** | **156535** | **3.0e-6** | **1.5e-8** | **0.4** | **0.5** | **0.4** | **-** | **0.91** |
|  | Secondary contact | -557.6 | 1125.3 | 39897 | 132588 | 151826 | 2.7e-6 | - | 0.3 | 0.41 | 0.48 | 0.79 | - |
|  | Ancient gene flow | -698.6 | 1407.3 | 85210 | 2081571 | 160629 | 4.6e-7 | - | 0.9 | 0.073 | 0.33 | 0.55 | - |
| T4CBHE-T5CBHE | Isolation | -490.1 | 986.3 | 68547 | 104808 | 62693 | - | - | - | - | 0.14 | - | - |
|  | Homo. sym. gene flow | -387.6 | 783.3 | 23383 | 100675 | 56530 | 3.6e-6 | - | 0.3 | 0.1 | 0.49 | - | - |
|  | **Hetero sym. gene flow** | **-383.8** | **779.6** | **32228** | **91660** | **58706** | **4.2e-6** | **1.5e-8** | **0.39** | **0.25** | **0.42** | **-** | **0.8** |
|  | Secondary contact | -387.1 | 784.3 | 37607 | 117434 | 58976 | 3.4e-6 | - | 0.40 | 0.20 | 0.37 | 0.28 | - |
|  | Ancient gene flow | -387.5 | 785.1 | 15850 | 96531 | 56065 | 3.6e-6 | - | 0.35 | 0.20 | 0.54 | 0.0023 | - |
| T1OCCH-T3OCCH | Isolation | -398.7 | 803.4 | 57914 | 117797 | 127973 |  |  | - | - | 0.34 | - | - |
|  | Homo. sym. gene flow | -316.7 | 641.5 | 28294 | 121020 | 114345 | 9.4e-7 | - | 0.11 | 0.10 | 0.53 | - | - |
|  | **Hetero sym. gene flow** | **-311.3** | **634.7** | **26159** | **119046** | **113869** | **1.3e-6** | **8.8e-8** | **0.16** | **0.15** | **0.54** | **-** | **0.82** |
|  | Secondary contact | -316.3 | 642.5 | 318801 | 1301633 | 1225057 | 9.4e-8 | - | 0.12 | 0.11 | 5.43 | 4.9 | - |
|  | Ancient gene flow | -314.1 | 638.3 | 20203 | 115644 | 108989 | 1.4e-6 | - | 0.16 | 0.15 | 0.58 | 0.039 | - |
| T1ONMO-T3ONMO | Isolation | -282.9 | 571.8 | 82049 | 165288 | 165953 | - | - | - | - | 0.44 | - | - |
|  | Homo. sym. gene flow | -208.9 | 425.8 | 27203 | 142309 | 147927 | 9.8e-7 | - | 0.13 | 0.14 | 0.82 | - | - |
|  | **Hetero sym. gene flow** | **-195.7** | **403.4** | **15843** | **143123** | **148824** | **5.4e-7** | **5.4e-8** | **0.078** | **0.080** | **0.89** | **-** | **0.78** |
|  | Secondary contact | -208.8 | 427.7 | 27172 | 142303 | 147987 | 9.8e-7 | - | 0.13 | 0.14 | 0.82 | 1.35 | - |
|  | Ancient gene flow | -282.9 | 575.8 | 82044 | 164704 | 166567 | 7.3e-9 | - | 0.0012 | 0.0012 | 0.44 | 0.74 | - |
| T1CBLM-T5CBLM | Isolation | -116.7 | 239.4 | 34289 | 111583 | 89107 | - | - | - | - | 0.61 | - | - |
|  | **Homo. sym. gene flow** | **-115.5** | **239.1** | **27330** | **110822** | **89510** | **6.2e-8** | **-** | **0.0068** | **0.0056** | **0.66** | **-** | **-** |
|  | **Hetero sym. gene flow** | **-115.5** | **243.0** | **27124** | **110669** | **89702** | **7.3e-8** | **1.8e-8** | **0.0081** | **0.0066** | **0.66** | **-** | **0.72** |
|  | **Secondary contact** | **-115.5** | **241.0** | **27786** | **111005** | **89654** | **6.4e-8** | **-** | **0.0071** | **0.0058** | **0.65** | **0.52** | **-** |
|  | **Ancient gene flow** | **-115.5** | **241.0** | **24892** | **109891** | **89328** | **1.1e-7** | **-** | **0.012** | **0.010** | **0.67** | **0.065** | **-** |
| T1CBHE-T5CBHE | Isolation | -142.1 | 290.2 | 52373 | 99970 | 92533 | - | - | - | - | 0.64 | - | - |
|  | **Homo. sym. gene flow** | **-132.1** | **272.2** | **9243** | **99915** | **93050** | **1.8e-7** | **-** | **0.018** | **0.017** | **0.92** | **-** | **-** |
|  | **Hetero sym. gene flow** | **-132.0** | **276.1** | **8282** | **100038** | **93345** | **2.4e-7** | **6.0e-8** | **0.024** | **0.022** | **0.92** | **-** | **0.72** |
|  | **Secondary contact** | **-131.9** | **273.9** | **16304** | **100999** | **93885** | **1.9e-7** | **-** | **0.019** | **0.017** | **0.87** | **0.54** | **-** |
|  | **Ancient gene flow** | **-132.1** | **274.1** | **34** | **54600** | **50800** | **1.9e-7** | **-** | **0. 0187** | **0.0174** | **0.527** | **0.051** | **-** |

**Supplementary Table 5:** Results of Partial Redundancy Analysis (pRDA) presenting the relative contribution of each environmental variable to the variation in species abundances across sampling sites conditioned on the reef.

| Environmental variable | Df | Variance | F | Pr(>F) | Significance |
| --- | --- | --- | --- | --- | --- |
| Temperature mean | 1 | 0.0041 | 37.98 | 0.001 | *** |
| Water clarity mean | 1 | 0.00059 | 5.38 | 0.001 | *** |
| Light intensity mean | 1 | 0.00032 | 2.92 | 0.005 | ** |
| Depth | 1 | 0.00030 | 2.81 | 0.002 | ** |
| Current speed mean | 1 | 0.00033 | 3.07 | 0.003 | ** |
| Current speed median | 1 | 0.00094 | 8.64 | 0.001 | *** |
| Residuals | 355 | 0.039 |  |  |  |
